# Identification of genetic association between cardiorespiratory fitness and the trainability genes in childhood acute lymphoblastic leukemia survivors

**DOI:** 10.1186/s12885-019-5651-z

**Published:** 2019-05-14

**Authors:** Maxime Caru, Kateryna Petrykey, Simon Drouin, Patrick Beaulieu, Pascal St-Onge, Valérie Lemay, Laurence Bertout, Caroline Laverdiere, Gregor Andelfinger, Maja Krajinovic, Daniel Sinnett, Daniel Curnier

**Affiliations:** 1 Laboratoire de Physiopathologie de l’EXercice (LPEX), École de Kinésiologie et des Sciences de l’Activité physique, Faculté de Médecine, Université de Montréal, CEPSUM, 2100, boulevard Édouard Montpetit, Montréal, QC H3C 3J7 Canada; 2 Department of psychology, Laboratoire EA 4430 – Clinique Psychanalyse Developpement (CliPsyD), University of Paris Nanterre, Nanterre, Ile-de-France France; 3 Research Center, Sainte-Justine University Health Center, Montreal, Quebec Canada; 4 Department of pharmacology and physiology, University of Montreal, Montreal, Quebec Canada; 5 Department of Pediatrics, University of Montreal, Montreal, Quebec Canada

**Keywords:** Acute lymphoblastic leukemia, Pediatric cancer survivorship, Genetic association study, Cardiorespiratory fitness, Cardiovascular health, Trainability genes

## Abstract

**Background:**

The progress of treatments of childhood acute lymphoblastic leukemia (ALL) has made it possible to reach a survival rate superior to 80%. However, the treatments lead to several long-term adverse effects, including cardiac toxicity. Although studies have reported associations between genetic variants and cardiorespiratory fitness, none has been performed on childhood ALL survivors.

**Methods:**

We performed whole-exome sequencing in 239 childhood ALL survivors from the PETALE cohort. Germline variants (both common and rare) in selected set of genes (*N* = 238) were analyzed for an association with cardiorespiratory fitness.

**Results:**

Our results showed that the common variant in the TTN gene was significantly associated with a low cardiorespiratory fitness level (*p* = 0.0005) and that the LEPR, IGFBPI and ENO3 genes were significantly associated with a low cardiorespiratory fitness level in female survivors (*p* ≤ 0.002). Also, we detected an association between the low cardiorespiratory fitness level in participants that were stratified to the “high risk” prognostic group and functionally predicted rare variants in the *SLC22A16* gene (*p* = 0.001). Positive associations between cardiorespiratory fitness level and trainability genes were mainly observed in females.

**Conclusions:**

For the first time, we observed that low cardiorespiratory fitness in childhood ALL survivors can be associated with variants in genes related to subjects’ trainability. These findings could allow better childhood ALL patient follow-up tailored to their genetic profile and cardiorespiratory fitness, which could help reduce at least some of the burden of long-term adverse effects of treatments.

**Electronic supplementary material:**

The online version of this article (10.1186/s12885-019-5651-z) contains supplementary material, which is available to authorized users.

## Background

Childhood acute lymphoblastic leukemia (ALL) is the most common pediatric cancer, representing about 30% of all childhood malignancies. Over the past four decades, the progress in treatments for childhood ALL has made it possible to achieve a five-year survival rate over 80% [1].

However, the aggressive treatments regimen often lead to several long-term adverse effects, including cardiac toxicity and cardiovascular issues in survivors [2]. Physical activity has an important role in the prevention of cardiovascular diseases and in the management of metabolic disorders. A good cardiorespiratory fitness level in childhood ALL survivors has shown a significant positive impact on mental health, quality of life, bone strength and mortality [3–6]. It has been shown in healthy and untrained populations that there is a great inter-individual variability in subjects’ capacity to improve their cardiac profile in response to regular exercise [7]. This can partly be explained by genetic variations between subjects, which can ultimately have an influence on their trainability [8]. Studies on healthy subjects and athletes have reported associations between genetic variants and changes in muscle strength [9, 10] and cardiorespiratory fitness in response to exercise [11]. It is essential to improve our knowledge of genetic determinants of trainability in childhood ALL survivors given that anticancer drugs, such as anthracyclines, can modify gene expression [12] and induce an impairment of cardiac function [13].

In this study, we investigated candidate genes linked to health-related fitness and performance and their association to high and low cardiorespiratory fitness levels in a cohort of childhood ALL survivors. Such genetic biomarkers will be important in the post-treatment follow-up of childhood ALL survivors by allowing personalized cardiorespiratory fitness risk management interventions.

## Methods

### Study population

All 239 participating childhood ALL survivors were diagnosed between 1987 and 2010 and treated according to DFCI-ALL 87–01 to 05–01 protocols [14] at Sainte-Justine University Health Center (SJUHC), Montreal (Quebec), Canada. The participants were recruited in the context of the PETALE study, a multidisciplinary research project with the goal to identify and to comprehensively characterize associated predictive biomarkers of long-term treatment related complications in childhood ALL survivors [15]. These participants had no history of refractory or recurrent disease and did not receive a hematopoietic stem cell transplant. These participants were almost exclusively of French Canadian descent (> 95%), with predominantly European ancestry [15]. In the current study, we restricted participants to those that were less than 19 years old at diagnosis. Subjects who have suffered from congenital bone disease or who received osteotoxic drugs for non-ALL disease were excluded. Written informed consent was obtained from every patient or parent/legal guardian. The study was conducted in accordance with the Declaration of Helsinki and the protocol was approved by the Ethics Review Committee of SJUHC.

### Measurements

#### Cardiopulmonary exercise test

The McMaster incremental cycle protocol [16] was performed on an electromagnetic cycle ergometer (Ergoline, ER900, Bitz, Germany). The test consisted of a standard incremental procedure at a pedaling cadence of 50–70 rpm starting at 25 W, increasing the load by 25 W or 50 W (depending on the height and sex of the subject) every 2 min. At the end of the test, an active rest period at 25 W for 3 min was followed by a passive rest period of 3 min. All subjects were monitored continuously during the test with twelve-lead electrocardiograms (CASE Exercise Testing, GE Marquette, Milwaukee, WI, USA). Blood pressure was measured every 2 min (Tango M2, SunTech Medical, Morrisville, NC, USA). Both the cardiologist and the exercise physiologist determined whether the subjects reached two out of three of the following criteria to validate their $\dot{\mathrm{V}}$ O_2_ peak: a respiratory exchange ratio value > 1.15, OMNI > 7, and HR_max_ ≥ 85% of the predicted value [17].

#### Predicted maximum oxygen uptake

The maximum oxygen uptake ($\dot{\mathrm{V}}\mathrm{O}2$ peak) was measured during the McMaster incremental cycle protocol with a breath-by-breath system (Oxycon Pro, Viasys Healthcare, Germany) calibrated prior to each test. Breath-by-breath data were averaged in 20 s intervals. For subjects ≥18 years old, predicted $\dot{\mathrm{V}}$ O_2_ peak was calculated using equations published by Wasserman and Hansen [18, 19], depending on age, sex, weight and height. For subjects < 18 years old the Cooper formula [19, 20] was used to predict $\dot{\mathrm{V}}$ O_2_ peak. An adjustment for overweight subjects was achieved with an addition of 6 mL.kg^− 1^.min^− 1^ for each kg of weight exceeding their normal weight [19] as calculated based on the 50th percentile of the World Health Organization body mass index charts for children [21].

#### Cardiorespiratory fitness level

The percentage of cardiorespiratory fitness is the ratio between measured and predicted $\dot{\mathrm{V}}$ O_2_peak: [($\dot{\mathrm{V}}$ O_2_ peak measured / $\dot{\mathrm{V}}$ O_2_ peak predicted) × 100. A higher percentage of $\dot{\mathrm{V}}$ O_2_ peak thus corresponded to a higher level of cardiorespiratory fitness.

### Whole Exome Sequencing (WES)

We performed whole-exome sequencing (WES) on a total of 239 participants. Whole exomes were captured in solution with Agilent’s SureSelect Human All Exon 50 Mb kits. Sequencing data (mean coverage ~40X) were generated on the SOLiD 4.0 (ThermoFisher Scientific) or Illumina HiSeq 2500 platform (mean coverage = 113.1X) (SJUHC and Génome Québec Integrated Centre for Pediatric Clinical Genomic platform). Reads were aligned on the Hg19 reference genome. PCR duplicates are removed using Picard [22]. Variant quality score recalibration was performed using the HaplotypeCaller implemented in Genome Analysis ToolKit (GATK) [23] and QC Failure reads was removed. Generic alignment format SAMtools was used to create pileup files from cleaned BAM files [24]. Rare and common variants with a predicted functional impact on protein were identified by functional annotation from ANNOVAR [25]. Variants considered as potentially damaging as per SIFT (< 0.1) [26] and Polyphen2 (≥0.85) [27]. Minor allele frequencies (MAF) were derived from the 1000 Genomes (European population) [28] and the NHLBI GO Exome Sequencing Project (European population, ESP) [29].

### Power analysis

Quanto software (version 1.2.4) [30] was used to compute a power analysis at 80%. For common variants (MAF of 5–30%), the power analysis revealed that the odds ratio ranged from 3 to 9 (depending on the outcome analyzed). The lowest odds ratio for rare variants (MAF of 0.01) that could be detected with a given sample size, was 26. The Bonferroni correction was used for the number of SNPs or genes tested.

### Association studies and statistical analysis

A list of candidate genes was generated through a review of the literature. The genes expected to influence the outcome associated with health-related fitness and performance (i.e. cardiorespiratory endurance, anaerobic, trainability, exercise hemodynamic, exercise intolerance and muscular strength) were targeted [31–33]. Additional candidate genes were selected via a Gene Oncology Enrichment Analysis and Visualization Tool (*Gorilla*) [34]. A total of 238 candidate genes (134 common variants and 1225 rare variants) were chosen (Additional file 1: Tables S1 and S2). Variants that did not conform to the Hardy-Weinberg equilibrium (*p* = 0.001) and/or with a total missing rate > 20% were excluded prior to analyses. Variants with a high linkage disequilibrium were excluded (*r*^2^ > 0.8).

Genetic association analyses between common genetic variants and cardiorespiratory fitness level were performed using allelic ratio implemented in PLINK v.1.07 [35]. The Benjamini and Hochberg method (FDR) was used to correct for multiple testing for candidate genes and variants with a FDR less than 0.10 were kept for further analyses [36–38]. Analyses were done by chi-square and Fisher’s exact test, as was applicable. Analyses were performed in all 239 subjects and upon stratification by sex, age at visit, time from the end of the treatment and prognostic risk group (i.e. standard risk (SR), high risk with and without cardio-protective agent dexrazoxane (HR and HR + DEX)) [14]. The cumulative doxorubicin dose for the SR group was 60 mg/m^2^, 300 mg/m^2^ for the HR + DEX group and either 360 mg/m^2^ (DFCI-ALL protocols 85–01, 87–01 and 91–01) or 300 mg/m^2^ (DFCI-ALL protocols 95–01) for the HR group. For rare variants associations, we used the SKAT-O test (Optimal Sequence Kernel Association Test) implemented in R v.3.2.1 [37, 39]. To test the individual contribution, we used the collapsing approach with the iterative exclusion of individual variants for rare variants associations. Adjustment for multiple testing was performed by bootstrap FDR method [40] implemented in QVALUE [41]. Genetic variants associated with cardiorespiratory fitness were analyzed using logistic regression including four covariables: age at visit, sex, cumulative doxorubicin dose and time from the end of the treatment.

For clinical characteristics, a two-tailed Student t-test with a significance level of 5% was performed. A Chi-square test with a threshold of 5% was used to compare two percentages. A Fisher exact test was used when the conditions for applying a Chi-square test were not met. All variables were reported as mean ± standard deviation (SD) or as percentage. Cardiorespiratory fitness data were verified for normal distribution using the Shapiro-Wilk test. All analyses were performed using SPSS version 24.0 [42].

## Results

A total of 239 childhood ALL survivors were included in our analyses (Table 1). Participants were divided in two groups according to their median predicted $\dot{\mathrm{V}}$ O_2_ peak. The first group opposed above and below the median of cardiorespiratory fitness level of the childhood ALL survivors (< 83.8% vs ≥83.8%) (Table 2), whereas the second group opposed the two extremes at least two standard deviations away from the median (≤48.3% vs ≥125.6%) (Additional file 1: Table S3).

**Table 1:** Clinical characteristics of acute lymphoblastic leukemia survivors

	Childhood ALL survivors	Females	Males
Total	239	120	119
Age at visit (y)	21.5 ± 6.1	21.5 ± 6.3	21.6 ± 6.0
Age at cancer diagnosis (y)	6.3 ± 4.6	6.2 ± 4.4	6.4 ± 4.7
Time from the end of the treatment (y)	13.0 ± 5.0	13.0 ± 5.3	12.9 ± 4.8
Weight (kg)	66.8 ± 16.4	63.7 ± 16.6	70.0 ± 15.7
Height (cm)	166.9 ± 10.0	160.3 ± 6.7	171.5 ± 9.6
$\dot{\mathrm{V}}$O_2_ peak (mL.kg^− 1^.min^− 1^)	32.6 ± 8.4	27.6 ± 6.5	36.9 ± 7.4*
Power at $\dot{\mathrm{V}}$ O_2_ peak (W)	174.7 ± 55.3	133.5 ± 28.9	213.8 ± 45.0*
$\dot{\mathrm{V}}$O_2_ peak predicted (%)	85.7 ± 17.1	86.8 ± 18.3*	83.2 ± 15.4
Physical activity (min)	27.8 ± 29.4	24.9 ± 30.3	30.5 ± 28.4*
DOX (mg/m^2^)	182.8 ± 119.7	183.4 ± 121.9	182.2 ± 117.9
DEX (mg/m^2^)	2781.0 ± 440.0	2828.8 ± 374.4	2731.8 ± 499.4

*ALL* acute lymphoblastic leukemia, *DOX* cumulative dose of doxorubicin, *DEX* cumulative dose of dexrazoxane. Physical activity variable represents minutes per day of moderate or intense leisure physical activities. Data are expressed as percentage or as means ± SD. *p < 0.05, Females versus Males

**Table 2:** Clinical characteristics of acute lymphoblastic leukemia survivors according to their cardiorespiratory fitness level

	Group 1	
	< 83.8%	≥83.8%
Total	119	120
Gender (Females / Males)	54/65	66/54
Age at visit (y)	21.6 ± 5.9	21.5 ± 6.4
Age at cancer diagnosis (y)	6.2 ± 4.8	6.3 ± 4.4
Time from the end of the treatment (y)	13.1 ± 5.1	12.8 ± 5.0
Weight (kg)	67.4 ± 18.1	66.2 ± 14.6
Height (cm)	166.1 ± 11.5	165.6 ± 8.3
$\dot{\mathrm{V}}$O_2_ peak (mL.kg^−1^.min^−1^)	28.5 ± 6.2	35.8 ± 8.6*
Power at $\dot{\mathrm{V}}$ O_2_ peak (W)	162.6 ± 55.2	185.6 ± 53.3*
$\dot{\mathrm{V}}$O_2_ peak predicted (%)	71.9 ± 10.0	98.0 ± 11.7*
Physical activity (min)	22.4 ± 29.6	32.9 ± 28.5*

Physical activity variable represents minutes per day of moderate or intense leisure physical activities. Data are expressed as percentages or as means ± SD. *p < .05

The common variants analyses are presented in Table 3 (also see Additional file 1: Table S4). The carriers of the *TTN* minor allele were overrepresented among survivors with a cardiorespiratory fitness level < 83.8%. The major allele in other genes (*LEPR, IGFBP1* and *ENO3*) had, in contrast, a protective role and were represented less frequently among female survivors who had cardiorespiratory fitness level < 83.8%. Significant genetic variants were further analyzed in logistic regression (Table 3 and Additional file 1: Table S4) in which the effect of genetic variant is adjusted for age at visit, sex, cumulative doxorubicin dose and time from end of the treatment and no significant confounding factors were observed in these associations. The analysis of functionally predicted rare variants detected an association (with FDR < 10%) between the low cardiorespiratory fitness level and rare variants enrichment in the *SLC22A16* gene for the HR group (*p* = 0.001) (Additional file 1: Table S5). Our analyses of rare variants showed a significant association with a low cardiorespiratory fitness in the HR group without the cardio-protective agent dexrazoxane. No other association with the appropriate FDR threshold was shown in our analysis of rare variants. In order to identify the individual contributions to the observed association signal, we explored different variant combinations with the collapsing approach. The significant signal came from the *SLC22A16* gene where three variants (rs41288594, rs61729086, rs146329765) were contributing to the signal (Additional file 1: Table S5). Our analyses reported the individual contribution of variant rs61729086 but showed no individual contributions of variants rs41288594 and rs146329765. The significant genetic variant was further analyzed in a logistic regression (Additional file 1: Table S5). Analyses revealed that there was no significant effect of co-variables in the multivariate model due to the confounding effects of other covariates and the small sample size of the low frequency genetic variant.

**Table 3:** Significant genetic associations between cardiorespiratory fitness and common variants

	Gene	SNPs ID	Minor Alleles		Major Alleles		*p*-value	FDR	MAF	OR (95% CI)	*p*-value*	OR (95% CI)*
			Affected	Unaffected	Affected	Unaffected						
			N (%)	N (%)	N (%)	N (%)						
Survivors < 83.8% predicted $\dot{\mathrm{V}}$ O2 peak	TTN	rs6723526	33 (14.47)	195 (85.53)	9 (4.55)	189 (95.45)	0.0005	0.04	0.1	3.55 (1.66–7.63)	0.0006	4.47 (1.89–10.53)
Female survivors < 83.8% predicted $\dot{\mathrm{V}}$ O2 peak	LEPR	rs1137100	22 (17.19)	106 (82.81)	34 (37.78)	56 (62.22)	0.0008	0.03	0.25	0.34 (0.18–0.64)	0.003	0.33 (0.15–0.68)
	IGFBP1	rs4619	43 (33.59)	85 (66.41)	51 (56.67)	39 (43.33)	0.0008	0.03	0.42	0.39 (0.22–0.67)	0.01	0.46(0.24–0.86)
	ENO3	rs238239	41 (32.54)	85 (67.46)	46 (53.49)	40 (46.51)	0.002	0.05	0.4	0.42 (0.24–0.74)	0.005	0.34 (0.16–0.73)

Threshold of FDR (false discovery rate) was 0.10. Affected were survivors who have a cardiorespiratory fitness level ≥ 83.8% and unaffected were survivors who have a cardiorespiratory fitness level < 83.8%. Results from genetic association were presented using allelic model, while results from adjusted logistic regression was presented using the genotypic model (i.e. additive)

*SNPs* single-nucleotide polymorphisms, *MAF* Minor allele frequency, *OR* Odds Ratio

*Logistic regression adjusted for age at visit, sex, cumulative doxorubicin dose and time from end of the treatment

## Discussion

This study was the first to assess genetic determinants of the cardiorespiratory fitness level in childhood ALL survivors. We observed that a low cardiorespiratory fitness was associated with genes primarily related to survivors’ trainability, particularly with common variants in the *TTN, LEPR, IGFBPI* and *ENO3* genes and with a rare variant in the *SLC22A16* gene. This was particularly obvious among female survivors.

The involvement of *TTN* and *LEPR*, two cardiac-related genes were associated with a decrease of cardiorespiratory fitness in survivors. Our findings showed that the common variant rs6723526 in *TTN* was associated to survivors with cardiorespiratory fitness level < 83.8%. *TTN*, which encodes the protein titin, one of the most abundant proteins in the human striated muscle myofilament [43] and plays an essential role in skeletal and cardiac muscle [44]. The HERITAGE family study demonstrated a similar influence of the *TTN* gene in regards to cardiac function during endurance training [45]. We also found that the common variant rs1137100 in the LEPR gene encoding the leptin receptor involved in body weight regulation [46], insulin resistance [47] and physiological adaptation response to exercise [48], was associated with a cardiorespiratory fitness level < 83.8% and ≤ 48.3% in female survivors. The *LEPR* gene had been reported by the HERITAGE study as associated with a lower $\dot{\mathrm{V}}$ O_2_ max training response [49] and was also associated with significantly lower physical activity levels in young children in another report [50]. Thus, the *TTN* and the *LEPR* genes are respectively associated with cardiac function during endurance training and a lower $\dot{\mathrm{V}}$ O_2_ max training response.

Another gene linked to insulin resistance is *IGFBP1*. This gene is also associated with lower cardiorespiratory fitness in female survivors. Indeed, we found that the common variant rs4619 in *IGFBP1* was associated with a cardiorespiratory fitness level < 83.8% and ≤ 48.3%, especially in female survivors. This gene is a member of the insulin-like growth factor binding protein family which plays an important role in cell migration and metabolic processes, influencing cardiorespiratory fitness level due to its role in energy metabolism [51]. It has been reported that while there is no significant change in *IGFBP1* levels in response to exercises with high resistance [52], changes are observed in response to exercises that are short in duration [53].

Similarly to the positive association between the expression of the IGFBP1 gene and physical inactivity [54], the enolase 3 (ENO3) gene is positively associated with exercise intolerance [55]. The *ENO3* gene is especially important because of its role in muscle development and regeneration [56]. It encodes β-enolase and is mainly expressed in adult striated muscle (i.e. skeletal and cardiac muscle) [57, 58]. It has been found that the absolute cellular level of enolase activity is higher in skeletal muscle than in other tissues (e.g., liver, kidney, spleen and neural tissues) [59] due to the heavy metabolic requirement of skeletal muscle [55]. Our findings showed that the common variant rs238239 in the *ENO3* gene was associated with a cardiorespiratory fitness level < 83.8% in female survivors. *LEPR*, *IGFBP1* and *ENO3* genes were found to be only significantly associated with a lower cardiorespiratory fitness in female survivors. As discussed previously, these genes play important roles in energy metabolism, body weight regulation and muscle development and regeneration [46, 51, 56]. Female survivors showed a greater impairment of total daily energy expenditure after treatments with anthracyclines [2]. Indeed, it is reported that in ALL survivors, cardiorespiratory levels are significantly lower in comparison to a similarly aged non-cancer population, especially in female survivors [60]. Many studies have reported a deterioration of the cardiorespiratory fitness in female survivors [60–63], which is consistent with our genetic association.

The absence of results in the common variants analysis with stratification by prognostic risk group (i.e. SR, HR and HR + DEX) was unexpected and could be due to lack of power. Indeed, several studies have shown genetic contributions to cardiac toxicity induced by higher cumulative dose of anthracyclines in pediatric cancer patients [64–66]. These findings confirmed that the doxorubicin dose can be an important risk factor [65, 67] which can affect the cancer subjects’ trainability [68] and their cardiovascular response [69–71]. It has been shown that the *SLC22A16* gene could contribute to variations in the doxorubicin pharmacokinetics, resulting in a possible higher incidence of adverse effects [72]. *SLC22A16* also plays a key role in mitochondrial fat oxidation by transporting carnitine, which is important during exercise [73]. Previous studies have shown that its supplementation of carnitine has the potential to improve β-oxidation during exercise in order to improve performance by making ATP available for mechanical work [74–77]. In this study, we found that a low cardiorespiratory fitness was detected in association with the presence of rare variants in the *SLC22A16* gene. Our findings revealed that there was no significant effect of co-variables in the multivariate model due to the confounding effects of other covariates. A previous study reported that the *SLC22A16* gene was associated with the doxorubicin dose in female cancer patients [78]. This could explain that for survivors in our HR group, the *SLC22A16* gene was negatively associated with cardiorespiratory fitness.

It should be noted that our statistical power is limited by the relatively small sample size (*n* = 239). The strengths of our study is the analysis of French Canadian population because it is characterized by its homogeneity, not only at the genetic level but also in terms of lifestyle [15]. Moreover, no significant differences between the frequency of common variant alleles between French-Canadian and other populations of European descent have been reported [79].

## Conclusion

In conclusion, this study provided evidence of genetic predispositions to low cardiorespiratory fitness level in childhood ALL survivors. However, further replication analysis and confirmation of these findings are needed. The identification of novel genetic biomarkers associated with lower cardiorespiratory fitness potential will help reduce the burden of cardiorespiratory long-term adverse effects. As a matter of fact, it will enable the development of personalized follow-ups and preventive strategies for childhood ALL survivors to mitigate this vulnerability.

## Additional file

Additional file 1: **Table S1.** List of common candidate genes included in the study. **Table S2.** List of rare candidate genes included in the study. **Table S3.** Clinical characteristics of acute lymphoblastic leukemia survivors with a very low or very high cardiorespiratory fitness level. **Table S4.** Significant genetic associations between very low or very high cardiorespiratory fitness and common variants. **Table S5.** Significant genetic associations between cardiorespiratory fitness and rare variants. (DOCX 101 kb)

## Abbreviations

ALL: Acute lymphoblastic leukemia
FDR: False discovery rate
HR: High risk without cardio-protective agent dexrazoxane
HR+DEX: High risk with cardio-protective agent dexrazoxane
MAF: Minor allele frequency
PETALE: Prévenir les effets tardifs des traitements de la leucémie aigüe lymphoblastique chez l’enfant
SNP: Single-nucleotide polymorphism
SR: Standard risk
$\dot{\mathrm{V}}$O_2_ peak: Maximal oxygen consumption (mL.kg^-1^.min^-1^)
WES: Whole exome sequencing
