# Supplementary material for: Identification of genetic association between cardiorespiratory fitness and the trainability genes in childhood acute lymphoblastic leukemia survivors
Source: BMC Cancer. 2019 May 14;19:443. doi: 10.1186/s12885-019-5651-z (PMC6515640; doi:10.1186/s12885-019-5651-z)
Supplement: Supplementary file 1 — Table S1. List of common candidate genes included in the study. Table S2. List of rare candidate genes included in the study. Table S3. Clinical characteristics of acute lymphoblastic leukemia survivors with a very low or very high cardiorespiratory fitness level. Table S4. Significant genetic associations between very low or very high cardiorespiratory fitness and common variants. Table S5. Significant genetic associations between cardiorespiratory fitness and rare variants. (DOCX 101 kb) [file 12885_2019_5651_MOESM1_ESM.docx]

Supplemental Digital Content, Table S1. List of common candidate genes included in the study

| **Genes** | **SNPs ID** | **Position** |
| --- | --- | --- |
| ABCB5 | rs34603556 | chr7:20691047-20691047 |
| ABCB5 | rs62453384 | chr7:20762646-20762646 |
| ABCB5 | rs61732039 | chr7:20687604-20687604 |
| ABCB8 | rs17545756 | chr7:150732812-150732812 |
| ABCC1 | rs45511401 | chr16:16173232-16173232 |
| ABCC3 | rs9890046 | chr17:48712711-48712711 |
| ABCC3 | rs12604031 | chr17:48712705-48712705 |
| ABCC3 | rs11568591 | chr17:48761053-48761053 |
| ABCC4 | rs2274407 | chr13:95859035-95859035 |
| ADD1 | rs4961 | chr4:2906707-2906707 |
| ADD1 | rs4963 | chr4:2916762-2916762 |
| AGT | rs4762 | chr1:230845977-230845977 |
| AKR1A1 | rs2229540 | chr1:46032311-46032311 |
| AKR1C3 | rs11551177 | chr10:5138747-5138747 |
| AMPD1 | rs61752479 | chr1:115231254-115231254 |
| APOE | rs440446 | chr19:45409167-45409167 |
| APOE | rs7412 | chr19:45412079-45412079 |
| ATF4 | rs4894 | chr22:39917515-39917515 |
| BDKRB2 | rs1046248 | chr14:96703484-96703484 |
| CASR | rs1042636 | chr3:122003769-122003769 |
| CBR1 | rs2835265 | chr21:37444696-37444696 |
| CBR1 | rs2835266 | chr21:37444697-37444697 |
| CBR3 | rs1056892 | chr21:37518706-37518706 |
| CETP | rs5880 | chr16:57015091-57015091 |
| COX15 | rs2231687 | chr10:101473218-101473218 |
| COX17 | rs2280578 | chr3:119395799-119395799 |
| CRTC1 | rs3746266 | chr19:18876309-18876309 |
| CYBA | rs4673 | chr16:88713236-88713236 |
| EDN1 | rs5370 | chr6:12296255-12296255 |
| ENO3 | rs238239 | chr17:4856580-4856580 |
| EPHX1 | rs1051740 | chr1:226019633-226019633 |
| ERBB2 | rs1058808 | chr17:37884037-37884037 |
| FABP2 | rs1799883 | chr4:120241902-120241902 |
| GABPB2 | rs11204774 | chr1:151062957-151062957 |
| GNB3 | rs5442 | chr12:6954864-6954864 |
| GSTM1 | rs147668562 | chr1:110231746-110231746 |
| HFE | rs1799945 | chr6:26091179-26091179 |
| HFE | rs1800562 | chr6:26093141-26093141 |
| HLA-A | rs3179982 | chr6:29912333-29912333 |
| HLA-A | rs2230954 | chr6:29910371-29910371 |
| HLA-A | rs707910 | chr6:29910663-29910663 |
| HLA-A | rs2231004 | chr6:29910762-29910762 |
| HLA-A | rs3173420 | chr6:29911114-29911114 |
| HLA-A | rs1059535 | chr6:29911222-29911222 |
| HLA-A |  | chr6:29911260-29911260 |
| HLA-A | rs76185201 | chr6:29911272-29911272 |

| **Genes** | **SNPs ID** | **Position** |
| --- | --- | --- |
| HLA-A | rs149288080 | chr6:29911925-29911925 |
| HLA-A | rs145046067 | chr6:29912030-29912030 |
| HLA-A | rs1136903 | chr6:29912280-29912280 |
| HLA-A | rs3180278 | chr6:29912345-29912345 |
| HLA-A | rs1137078 | chr6:29912348-29912348 |
| HLA-A | rs2231119 | chr6:29912856-29912856 |
| HLA-A | rs1137631 | chr6:29913037-29913037 |
| HLA-A | rs1136949 | chr6:29912297-29912297 |
| HLA-A | rs1137160 | chr6:29912386-29912386 |
| HLA-A | rs1136683 | chr6:29910752-29910752 |
| HLA-A | rs1136702 | chr6:29911092-29911092 |
| HLA-A | rs150028516 | chr6:29912042-29912042 |
| HLA-A | rs2230991 | chr6:29910719-29910719 |
| HLA-A | rs1059526 | chr6:29911218-29911218 |
| HLA-A | rs1136659 | chr6:29910557-29910557 |
| HLA-A | rs1059423 | chr6:29910581-29910581 |
| IGFBP1 | rs4619 | chr7:45932669-45932669 |
| LEPR | rs1137100 | chr1:66036441-66036441 |
| LEPR | rs1805094 | chr1:66075952-66075952 |
| LIPC | rs6083 | chr15:58838010-58838010 |
| LMNA | rs593987 | chr1:156096387-156096387 |
| LMNA | rs513043 | chr1:156099669-156099669 |
| LRP5 | rs4988321 | chr11:68174189-68174189 |
| LTBP4 | rs10880 | chr19:41128309-41128309 |
| NCOA3 | rs2230782 | chr20:46264888-46264888 |
| NDUFA10 | rs13848 | chr2:240946766-240946766 |
| NDUFA10 | rs1530144 | chr2:240923050-240923050 |
| NDUFA10 | rs11541494 | chr2:240964714-240964714 |
| NDUFA11 | rs12980262 | chr19:5893058-5893058 |
| NDUFA11 | rs1678868 | chr19:5892954-5892954 |
| NDUFA6 | rs1801311 | chr22:42486723-42486723 |
| NDUFS2 | rs11576415 | chr1:161182208-161182208 |
| NDUFS3 | rs2030166 | chr11:47602729-47602729 |
| NDUFV3 | rs10595 | chr21:44324365-44324365 |
| NOS2 | rs2297518 | chr17:26096597-26096597 |
| NOS3 | rs79467411 | chr7:150700291-150700291 |
| NQO1 | rs1800566 | chr16:69745145-69745145 |
| PFKM | rs11609399 | chr12:48501161-48501161 |
| PFKM | rs4760682 | chr12:48512285-48512285 |
| PFKM | rs11168414 | chr12:48512331-48512331 |
| PON1 | rs854560 | chr7:94946084-94946084 |
| PPARA | rs1800206 | chr22:46614274-46614274 |
| PPARG | rs1801282 | chr3:12393125-12393125 |
| PPARGC1A | rs3736265 | chr4:23814707-23814707 |
| SGCG | rs17314986 | chr13:23824818-23824818 |
| SIRT3 | rs28365927 | chr11:236091-236091 |
| **Genes** | **SNPs ID** | **Position** |
| SLC22A16 | rs12210538 | chr6:110760008-110760008 |
| SLC22A16 | rs723685 | chr6:110763875-110763875 |
| SLC28A3 | rs56350726 | chr9:86900369-86900369 |
| SLC28A3 | rs10868138 | chr9:86917301-86917301 |
| TOP1MT | rs2293925 | chr8:144392368-144392368 |
| TTN | rs3829747 | chr2:179397561-179397561 |
| TTN | rs3731749 | chr2:179406191-179406191 |
| TTN | rs9808377 | chr2:179421694-179421694 |
| TTN | rs3731746 | chr2:179430997-179430997 |
| TTN | rs744426 | chr2:179436020-179436020 |
| TTN | rs2303838 | chr2:179444939-179444939 |
| TTN | rs2042996 | chr2:179451420-179451420 |
| TTN | rs16866406 | chr2:179457147-179457147 |
| TTN | rs2288569 | chr2:179458591-179458591 |
| TTN | rs1001238 | chr2:179464527-179464527 |
| TTN | rs6723526 | chr2:179498042-179498042 |
| TTN | rs2042995 | chr2:179558366-179558366 |
| TTN | rs7585334 | chr2:179620951-179620951 |
| TTN | rs2291310 | chr2:179623758-179623758 |
| TTN | rs2291311 | chr2:179629461-179629461 |
| TTN | rs10497520 | chr2:179644855-179644855 |
| TTN | rs12463674 | chr2:179432185-179432185 |
| TTN | rs36051007 | chr2:179545859-179545859 |
| TTN | rs2244492 | chr2:179554305-179554305 |
| TTN | rs200875815 | chr2:179634421-179634421 |
| TTN | rs3829746 | chr2:179427536-179427536 |
| TTN | rs12693164 | chr2:179579093-179579093 |
| TTN | rs13390491 | chr2:179582327-179582327 |
| TTN | rs2627043 | chr2:179582537-179582537 |
| TTN | rs16866465 | chr2:179583496-179583496 |
| TTN | rs12693166 | chr2:179587130-179587130 |
| TTN | rs35813871 | chr2:179650408-179650408 |
| TTN | rs2627037 | chr2:179606538-179606538 |
| TTN | rs16866538 | chr2:179659912-179659912 |
| TTN | rs72648998 | chr2:179575511-179575511 |
| ULK1 | rs7138581 | chr12:132406666-132406666 |
| VDR | rs2228570 | chr12:48272895-48272895 |

Supplemental Digital Content, Table S2. List of rare candidate genes included in the study

| **Genes** | **SNPs ID** | **Position** |
| --- | --- | --- |
| ABCB1 |  | chr7:87179813-87179813 |
| ABCB1 |  | chr7:87214975-87214975 |
| ABCB1 | rs56107566 | chr7:87175288-87175288 |
| ABCB1 |  | chr7:87145953-87145953 |
| ABCB1 | rs2032582 | chr7:87160618-87160618 |
| ABCB1 |  | chr7:87196164-87196164 |
| ABCB1 | rs77543238 | chr7:87193460-87193460 |
| ABCB1 | rs55852620 | chr7:87138760-87138760 |
| ABCB1 | rs202002337 | chr7:87196161-87196161 |
| ABCB1 |  | chr7:87133704-87133704 |
| ABCB1 | rs201661522 | chr7:87168609-87168609 |
| ABCB1 | rs142600685 | chr7:87179256-87179256 |
| ABCB5 |  | chr7:20685488-20685488 |
| ABCB5 | rs151326874 | chr7:20691167-20691167 |
| ABCB5 | rs17143304 | chr7:20767954-20767954 |
| ABCB5 |  | chr7:20739510-20739510 |
| ABCB5 |  | chr7:20685493-20685493 |
| ABCB5 | rs150545949 | chr7:20683170-20683170 |
| ABCB5 | rs17143212 | chr7:20682884-20682884 |
| ABCB5 | rs2074000 | chr7:20685484-20685484 |
| ABCB5 | rs140251822 | chr7:20721260-20721260 |
| ABCB5 |  | chr7:20782528-20782528 |
| ABCB5 |  | chr7:20691184-20691184 |
| ABCB5 |  | chr7:20691185-20691185 |
| ABCB5 | rs60197951 | chr7:20784910-20784910 |
| ABCB5 |  | chr7:20698170-20698170 |
| ABCB5 |  | chr7:20768024-20768024 |
| ABCB5 |  | chr7:20739703-20739703 |
| ABCB8 | rs117793104 | chr7:150730966-150730966 |
| ABCC1 |  | chr16:16165569-16165569 |
| ABCC1 | rs60782127 | chr16:16142079-16142079 |
| ABCC1 | rs200039403 | chr16:16208889-16208889 |
| ABCC1 |  | chr16:16162142-16162142 |
| ABCC2 |  | chr10:101559093-101559093 |
| ABCC2 |  | chr10:101553324-101553324 |
| ABCC2 | rs45441199 | chr10:101591737-101591737 |
| ABCC2 | rs17222617 | chr10:101578952-101578952 |
| ABCC2 | rs17216317 | chr10:101604107-101604107 |
| ABCC2 | rs56131651 | chr10:101557063-101557063 |
| ABCC2 |  | chr10:101564013-101564013 |
| ABCC2 |  | chr10:101596043-101596043 |
| ABCC2 | rs145672804 | chr10:101591866-101591866 |
| ABCC2 |  | chr10:101577182-101577182 |
| ABCC3 |  | chr17:48746739-48746739 |
| ABCC3 |  | chr17:48741354-48741354 |
| ABCC3 | rs148982238 | chr17:48762223-48762223 |
| ABCC3 |  | chr17:48757208-48757208 |

| **Genes** | **SNPs ID** | **Position** |
| --- | --- | --- |
| ABCC3 |  | chr17:48765063-48765063 |
| ABCC3 |  | chr17:48755178-48755178 |
| ABCC3 | rs34502058 | chr17:48753045-48753045 |
| ABCC3 | rs41280128 | chr17:48761062-48761062 |
| ABCC3 |  | chr17:48744940-48744940 |
| ABCC3 | rs140690263 | chr17:48765024-48765024 |
| ABCC3 | rs143710549 | chr17:48762190-48762190 |
| ABCC3 |  | chr17:48742541-48742541 |
| ABCC3 |  | chr17:48744966-48744966 |
| ABCC3 |  | chr17:48755145-48755145 |
| ABCC3 |  | chr17:48746251-48746251 |
| ABCC4 | rs11568658 | chr13:95863008-95863008 |
| ABCC4 | rs11568644 | chr13:95705380-95705380 |
| ABCC4 |  | chr13:95696575-95696575 |
| ABCC4 |  | chr13:95829966-95829966 |
| ABCC4 |  | chr13:95705424-95705424 |
| ABCC4 | rs11568694 | chr13:95735520-95735520 |
| ABCC4 | rs367759893 | chr13:95830300-95830300 |
| ABCC4 |  | chr13:95860061-95860061 |
| ABCC4 |  | chr13:95818570-95818570 |
| ABCC5 |  | chr3:183681255-183681255 |
| ABCC5 |  | chr3:183669271-183669271 |
| ABCC5 |  | chr3:183677620-183677620 |
| ABCC5 |  | chr3:183677621-183677621 |
| ABCC5 | rs368112213 | chr3:183689395-183689395 |
| ABCC5 |  | chr3:183681203-183681203 |
| ABCC5 |  | chr3:183679412-183679412 |
| ABCC5 | rs369624798 | chr3:183700648-183700648 |
| ABCC5 |  | chr3:183695316-183695316 |
| ABCC8 |  | chr11:17418492-17418492 |
| ABCC8 |  | chr11:17414546-17414546 |
| ABCC8 |  | chr11:17418522-17418522 |
| ABCC8 |  | chr11:17448659-17448659 |
| ABCC8 |  | chr11:17432139-17432139 |
| ABCC8 |  | chr11:17432140-17432140 |
| ABCG2 | rs45605536 | chr4:89018670-89018670 |
| ABCG2 |  | chr4:89052317-89052317 |
| ABCG2 | rs199473672 | chr4:89052998-89052998 |
| ABCG2 |  | chr4:89016692-89016692 |
| ABCG2 |  | chr4:89016693-89016693 |
| ABCG2 |  | chr4:89016694-89016694 |
| ABCG2 |  | chr4:89060981-89060981 |
| ACADVL | rs113994168 | chr17:7125522-7125522 |
| ACADVL |  | chr17:7125611-7125611 |
| ACADVL,  DLG4 | rs78514016 | chr17:7123194-7123194 |
| ACE | rs200754517 | chr17:61574642-61574642 |
| **Genes** | **SNPs ID** | **Position** |
| ACE | rs3730025 | chr17:61557773-61557773 |
| ACE | rs374146846 | chr17:61566011-61566011 |
| ACE | rs4303 | chr17:61557823-61557823 |
| ACE | rs3730043 | chr17:61568577-61568577 |
| ACE | rs140941300 | chr17:61574311-61574311 |
| ACE |  | chr17:61566436-61566436 |
| ACE | rs143320537 | chr17:61556415-61556415 |
| ACE | rs141543325 | chr17:61557724-61557724 |
| ACE | rs142350301 | chr17:61559041-61559041 |
| ACE | rs148193919 | chr17:61559038-61559038 |
| ACE | rs200649158 | chr17:61564014-61564014 |
| ACE | rs147429960 | chr17:61562654-61562654 |
| ACE |  | chr17:61560518-61560518 |
| ACE |  | chr17:61574233-61574233 |
| ACE | rs144494842 | chr17:61559967-61559967 |
| ACO1 | rs113309164 | chr9:32436107-32436107 |
| ACO1 |  | chr9:32433782-32433782 |
| ACO1 | rs41304757 | chr9:32408612-32408612 |
| ACO1 |  | chr9:32434610-32434610 |
| ACO1 |  | chr9:32427386-32427386 |
| ACO1 |  | chr9:32430509-32430509 |
| ACO1 | rs147879556 | chr9:32430510-32430510 |
| ACO1 | rs41313772 | chr9:32405589-32405589 |
| ACO1 | rs61753543 | chr9:32418473-32418473 |
| ADD1 |  | chr4:2883668-2883668 |
| ADD1 |  | chr4:2906573-2906573 |
| ADD1 |  | chr4:2909532-2909532 |
| ADD1 |  | chr4:2930123-2930123 |
| ADD1 |  | chr4:2899954-2899954 |
| ADIPOR1 |  | chr1:202913044-202913044 |
| ADRA2A |  | chr10:112838136-112838136 |
| ADRA2A |  | chr10:112839111-112839111 |
| ADRA2A | rs1800035 | chr10:112838552-112838552 |
| ADRA2B |  | chr2:96780795-96780795 |
| ADRA2B | rs29000569 | chr2:96780763-96780763 |
| ADRB1 |  | chr10:115804233-115804233 |
| ADRB2 | rs373763314 | chr5:148206591-148206591 |
| ADRB3 |  | chr8:37823349-37823349 |
| ADRB3 | rs199753604 | chr8:37823431-37823431 |
| ADRB3 | rs201873040 | chr8:37823306-37823306 |
| AGT |  | chr1:230846581-230846581 |
| AGT | rs56073403 | chr1:230845755-230845755 |
| AGT |  | chr1:230840024-230840024 |
| AGTR1 |  | chr3:148459522-148459522 |
| AIFM2 | rs41277978 | chr10:71883836-71883836 |
| AIFM2 |  | chr10:71876524-71876524 |
| AIFM2 | rs146365906 | chr10:71876390-71876390 |
| AIFM2 |  | chr10:71874714-71874714 |
| AIFM3 |  | chr22:21330954-21330954 |
| AIFM3 |  | chr22:21328571-21328571 |
| **Genes** | **SNPs ID** | **Position** |
| AIFM3 | rs139501949 | chr22:21331032-21331032 |
| AIFM3 | rs367787216 | chr22:21333954-21333954 |
| AKR1A1 |  | chr1:46035585-46035585 |
| AKR1A1 | rs188867600 | chr1:46035584-46035584 |
| AKR1C3 |  | chr10:5141583-5141583 |
| AKR1C3 | rs34186955 | chr10:5141609-5141609 |
| AKR1C3 |  | chr10:5138710-5138710 |
| AKR1C3 | rs139011578 | chr10:5138672-5138672 |
| AKR1C3 | rs200981816 | chr10:5139642-5139642 |
| AMPD1 | rs34526199 | chr1:115222237-115222237 |
| AMPD1 |  | chr1:115231354-115231354 |
| AMPD1 | rs140181682 | chr1:115218260-115218260 |
| AMPD1 | rs61738827 | chr1:115229523-115229523 |
| AMPD1 | rs61752478 | chr1:115221116-115221116 |
| AMPD1 |  | chr1:115223070-115223070 |
| AMPD1 |  | chr1:115220982-115220982 |
| AMPD1 |  | chr1:115222328-115222328 |
| AMPD1 | rs139582106 | chr1:115226899-115226899 |
| AMPD1 |  | chr1:115220638-115220638 |
| AMPD1 |  | chr1:115222275-115222275 |
| APOA1 |  | chr11:116707747-116707747 |
| APOA1 |  | chr11:116706859-116706859 |
| APOC3 |  | chr11:116701271-116701271 |
| APOE | rs267606661 | chr19:45412358-45412358 |
| ATF4 | rs146469032 | chr22:39917949-39917949 |
| ATF4 | rs150385569 | chr22:39917960-39917960 |
| ATF4 | rs146633351 | chr22:39918175-39918175 |
| ATP1A2 | rs201048446 | chr1:160106787-160106787 |
| ATP1A2 |  | chr1:160093201-160093201 |
| ATP1A2 |  | chr1:160104295-160104295 |
| ATP1A2 |  | chr1:160095019-160095019 |
| ATP1A2 |  | chr1:160098598-160098598 |
| ATP1A2 | rs189922200 | chr1:160098688-160098688 |
| ATP2A2 | rs55984131 | chr12:110734406-110734406 |
| BDKRB2 |  | chr14:96706744-96706744 |
| BDKRB2 |  | chr14:96707774-96707774 |
| BDKRB2 | rs202134620 | chr14:96707173-96707173 |
| BDKRB2 |  | chr14:96707156-96707156 |
| CASQ2 |  | chr1:116310934-116310934 |
| CASQ2 | rs10801999 | chr1:116310937-116310937 |
| CASQ2 |  | chr1:116247824-116247824 |
| CASR |  | chr3:121973184-121973184 |
| CASR | rs200386687 | chr3:121980661-121980661 |
| CASR |  | chr3:121981072-121981072 |
| CASR | rs117375173 | chr3:122002576-122002576 |
| CASR |  | chr3:121976091-121976091 |
| CASR |  | chr3:122002740-122002740 |
| CAT | rs147298685 | chr11:34477610-34477610 |
| CAT |  | chr11:34470833-34470833 |
| CAT |  | chr11:34477621-34477621 |
| **Genes** | **SNPs ID** | **Position** |
| CAT |  | chr11:34477698-34477698 |
| CBR1 | rs41557318 | chr21:37443349-37443349 |
| CBR1 | rs35710857 | chr21:37444120-37444120 |
| CBR1 |  | chr21:37444971-37444971 |
| CBR1 |  | chr21:37445131-37445131 |
| CBR3 | rs16993929 | chr21:37510224-37510224 |
| CBR3 | rs370219967 | chr21:37518728-37518728 |
| CBR3 | rs144228729 | chr21:37507719-37507719 |
| CETP | rs34065661 | chr16:56995935-56995935 |
| CETP |  | chr16:57003419-57003419 |
| CFTR |  | chr7:117304758-117304758 |
| CFTR | rs1800076 | chr7:117149147-117149147 |
| CFTR |  | chr7:117243766-117243766 |
| CFTR | rs201880593 | chr7:117188750-117188750 |
| CFTR | rs74571530 | chr7:117199648-117199648 |
| CFTR |  | chr7:117144392-117144392 |
| CFTR | rs1800079 | chr7:117174349-117174349 |
| CFTR | rs113993959 | chr7:117227832-117227832 |
| CFTR | rs1800100 | chr7:117232223-117232223 |
| CFTR | rs1800110 | chr7:117243828-117243828 |
| CFTR | rs1800112 | chr7:117250664-117250664 |
| CFTR |  | chr7:117243752-117243752 |
| CFTR |  | chr7:117243753-117243753 |
| CFTR | rs121908752 | chr7:117175339-117175339 |
| CFTR | rs1800111 | chr7:117250575-117250575 |
| CFTR | rs1800073 | chr7:117144344-117144344 |
| CFTR |  | chr7:117267711-117267711 |
| CFTR | rs1800098 | chr7:117230454-117230454 |
| CFTR | rs1800086 | chr7:117180336-117180336 |
| CFTR |  | chr7:117232027-117232027 |
| CHRM2 |  | chr7:136700024-136700024 |
| CKM | rs11559024 | chr19:45821183-45821183 |
| CKM | rs17357122 | chr19:45815163-45815163 |
| CKM | rs145987658 | chr19:45815121-45815121 |
| CNTF |  | chr11:58391946-58391946 |
| COL1A1 |  | chr17:48267952-48267952 |
| COL1A1 |  | chr17:48276925-48276925 |
| COL1A1 | rs139593707 | chr17:48266141-48266141 |
| COL1A1 |  | chr17:48266784-48266784 |
| COL1A1 |  | chr17:48265305-48265305 |
| COL1A1 | rs147266928 | chr17:48263206-48263206 |
| COL1A1 |  | chr17:48264226-48264226 |
| COMT | rs13306279 | chr22:19951803-19951803 |
| COX10 | rs113058506 | chr17:14110489-14110489 |
| COX10 |  | chr17:14110264-14110264 |
| COX10 | rs145948285 | chr17:13980176-13980176 |
| COX10 |  | chr17:13977645-13977645 |
| COX10 |  | chr17:13977646-13977646 |
| COX10 |  | chr17:13980356-13980356 |
| COX11 |  | chr17:53045929-53045929 |
| **Genes** | **SNPs ID** | **Position** |
| COX11 |  | chr17:53040261-53040261 |
| COX11 |  | chr17:53040128-53040128 |
| COX15 |  | chr10:101478205-101478205 |
| COX15 |  | chr10:101491735-101491735 |
| COX15 | rs192078749 | chr10:101486775-101486775 |
| COX15 |  | chr10:101480780-101480780 |
| COX15 | rs138293000 | chr10:101478161-101478161 |
| COX5A |  | chr15:75219139-75219139 |
| COX5A |  | chr15:75219145-75219145 |
| COX5A | rs200367305 | chr15:75230282-75230282 |
| CPT2 | rs74315294 | chr1:53668099-53668099 |
| CPT2 |  | chr1:53676011-53676011 |
| CPT2 |  | chr1:53675923-53675923 |
| CPT2 |  | chr1:53666413-53666413 |
| CRTC1 | rs199510737 | chr19:18888195-18888195 |
| CYBA | rs114610092 | chr16:88709946-88709946 |
| CYP19A1 | rs700519 | chr15:51507968-51507968 |
| CYP19A1 |  | chr15:51503186-51503186 |
| CYP19A1 |  | chr15:51503187-51503187 |
| CYP19A1 | rs28757184 | chr15:51514572-51514572 |
| CYP19A1 | rs141305220 | chr15:51503159-51503159 |
| CYP19A1 | rs143562020 | chr15:51510760-51510760 |
| CYP19A1 |  | chr15:51514548-51514548 |
| CYP19A1 |  | chr15:51503084-51503084 |
| CYP19A1 |  | chr15:51503070-51503070 |
| DIO1 |  | chr1:54370401-54370401 |
| DIO1 |  | chr1:54360058-54360058 |
| DRD2 | rs1801028 | chr11:113283484-113283484 |
| DRD2 |  | chr11:113281635-113281635 |
| DRD2 |  | chr11:113285126-113285126 |
| EDN1 | rs149399492 | chr6:12292628-12292628 |
| EDN1 |  | chr6:12294568-12294568 |
| ENO3 | rs141103742 | chr17:4856119-4856119 |
| ENO3 | rs151067614 | chr17:4857090-4857090 |
| ENO3 |  | chr17:4858782-4858782 |
| ENPP1 |  | chr6:132189260-132189260 |
| ENPP1 | rs28933977 | chr6:132206079-132206079 |
| ENPP1 |  | chr6:132189254-132189254 |
| ENPP1 |  | chr6:132189263-132189263 |
| ENPP1 |  | chr6:132189258-132189258 |
| ENPP1 |  | chr6:132185680-132185680 |
| ENPP1 |  | chr6:132193231-132193231 |
| ENPP1 |  | chr6:132181558-132181558 |
| ENPP1 | rs142001296 | chr6:132172374-132172374 |
| ENPP1 |  | chr6:132203534-132203534 |
| ENPP1 | rs371695495 | chr6:132171244-132171244 |
| ENPP1 |  | chr6:132211506-132211506 |
| EPAS1 |  | chr2:46611678-46611678 |
| EPAS1 |  | chr2:46611677-46611677 |
| EPAS1 |  | chr2:46583926-46583926 |
| **Genes** | **SNPs ID** | **Position** |
| EPAS1 |  | chr2:46607490-46607490 |
| EPAS1 |  | chr2:46588037-46588037 |
| EPAS1 |  | chr2:46587880-46587880 |
| EPAS1 |  | chr2:46587881-46587881 |
| EPHX1 |  | chr1:226026938-226026938 |
| EPHX1 |  | chr1:226016566-226016566 |
| EPHX1 | rs148810909 | chr1:226027029-226027029 |
| EPHX1 |  | chr1:226030146-226030146 |
| ERBB2 | rs201021373 | chr17:37872115-37872115 |
| ERBB2 | rs55943169 | chr17:37884176-37884176 |
| ERBB2 |  | chr17:37866395-37866395 |
| ERBB2 |  | chr17:37884285-37884285 |
| ERBB2 |  | chr17:37884217-37884217 |
| ERBB2 | rs201470725 | chr17:37872806-37872806 |
| ERBB2 |  | chr17:37873672-37873672 |
| ERBB2 |  | chr17:37882019-37882019 |
| ERCC2 |  | chr19:45868386-45868386 |
| ERCC2 |  | chr19:45855769-45855769 |
| ERCC2 |  | chr19:45867268-45867268 |
| ERCC2 |  | chr19:45860581-45860581 |
| ERCC2 |  | chr19:45860928-45860928 |
| ESR1 | rs200075329 | chr6:152129399-152129399 |
| ESR1 | rs139960913 | chr6:152129063-152129063 |
| ESRRA | rs200986301 | chr11:64083221-64083221 |
| ESRRA | rs201336331 | chr11:64083269-64083269 |
| ESRRA | rs201971362 | chr11:64083293-64083293 |
| ESRRA | rs201072913 | chr11:64083320-64083320 |
| ESRRA | rs79204587 | chr11:64083328-64083328 |
| ESRRA | rs80310817 | chr11:64083331-64083331 |
| ETFDH |  | chr4:159606318-159606318 |
| FGA |  | chr4:155505855-155505855 |
| GABPB2 | rs62621412 | chr1:151089885-151089885 |
| GABPB2 |  | chr1:151065721-151065721 |
| GABPB2 |  | chr1:151060735-151060735 |
| GABPB2 |  | chr1:151070446-151070446 |
| GATA4 |  | chr8:11615875-11615875 |
| GATA4 | rs56208331 | chr8:11615928-11615928 |
| GATA4 |  | chr8:11615936-11615936 |
| GATA4 |  | chr8:11615955-11615955 |
| GNAS |  | chr20:57415535-57415535 |
| GNAS |  | chr20:57484756-57484756 |
| GNAS |  | chr20:57430261-57430261 |
| GNAS | rs148033592 | chr20:57429696-57429696 |
| GNAS | rs187415363 | chr20:57415366-57415366 |
| GNB3 |  | chr12:6954921-6954921 |
| GNB3 |  | chr12:6954802-6954802 |
| GNB3 |  | chr12:6954955-6954955 |
| GSTT1 |  | chr22:24381742-24381742 |
| GSTT1 | rs11550605 | chr22:24379402-24379402 |
| GYS1 |  | chr19:49477888-49477888 |
| **Genes** | **SNPs ID** | **Position** |
| GYS1 |  | chr19:49477914-49477914 |
| GYS1 |  | chr19:49490482-49490482 |
| GYS1 |  | chr19:49490557-49490557 |
| GYS1 | rs201548356 | chr19:49489119-49489119 |
| HAS3 | rs140561541 | chr16:69143315-69143315 |
| HAS3 |  | chr16:69143510-69143510 |
| HAS3 | rs142125256 | chr16:69152282-69152282 |
| HFE | rs1800730 | chr6:26091185-26091185 |
| HFE |  | chr6:26091188-26091188 |
| HIF1A |  | chr14:62200905-62200905 |
| HIF1A | rs41508050 | chr14:62204808-62204808 |
| HIF1A |  | chr14:62203788-62203788 |
| HIF1A |  | chr14:62199147-62199147 |
| HIF1A |  | chr14:62199148-62199148 |
| HIF1A |  | chr14:62204837-62204837 |
| HIF1A |  | chr14:62200906-62200906 |
| HIF1A | rs149348765 | chr14:62204819-62204819 |
| HIF1A |  | chr14:62203623-62203623 |
| HIF1A |  | chr14:62213727-62213727 |
| HIF1A |  | chr14:62207788-62207788 |
| HLA-A | rs1059488 | chr6:29911098-29911098 |
| HLA-A | rs9260157 | chr6:29911246-29911246 |
| HLA-A | rs3098019 | chr6:29911271-29911271 |
| HLA-A | rs41559916 | chr6:29911296-29911296 |
| HLA-A | rs1136741 | chr6:29911901-29911901 |
| HLA-A | rs1059563 | chr6:29911928-29911928 |
| HLA-A | rs1059632 | chr6:29912087-29912087 |
| HLA-A | rs1059896 | chr6:29912342-29912342 |
| HLA-A | rs1059516 | chr6:29911198-29911198 |
| HLA-A | rs1059520 | chr6:29911207-29911207 |
| HLA-A | rs41544012 | chr6:29910688-29910688 |
| HLA-A | rs41559716 | chr6:29910693-29910693 |
| HLA-A | rs9391665 | chr6:29911306-29911306 |
| HLA-A | rs199474424 | chr6:29910721-29910721 |
| HLA-A | rs45569434 | chr6:29910572-29910572 |
| HLA-A | rs1059449 | chr6:29910698-29910698 |
| HLA-A | rs1064588 | chr6:29910717-29910717 |
| HLA-A | rs2230991 | chr6:29910719-29910719 |
| HLA-A |  | chr6:29911114-29911114 |
| HLA-A | rs1059537 | chr6:29911227-29911227 |
| HLA-A | rs1137110 | chr6:29912373-29912373 |
| HLA-A | rs41552219 | chr6:29910602-29910602 |
| HLA-A | rs41559117 | chr6:29910660-29910660 |
| HLA-A | rs41558618 | chr6:29912301-29912301 |
| HLA-A | rs79326316 | chr6:29910340-29910340 |
| HLA-A | rs1059451 | chr6:29910699-29910699 |
| HLA-A | rs199474457 | chr6:29910750-29910750 |
| HLA-A | rs76185201 | chr6:29911272-29911272 |
| HLA-A | rs41554316 | chr6:29912315-29912315 |
| HLA-A | rs41545116 | chr6:29910566-29910566 |
| **Genes** | **SNPs ID** | **Position** |
| HLA-A | rs1059626 | chr6:29912085-29912085 |
| HLA-A | rs62687162 | chr6:29911320-29911320 |
| HLA-A | rs41563116 | chr6:29911284-29911284 |
| HLA-A | rs41557315 | chr6:29911908-29911908 |
| HLA-A | rs45585732 | chr6:29910549-29910549 |
| HLA-A | rs61760919 | chr6:29911267-29911267 |
| HLA-A | rs201603558 | chr6:29910627-29910627 |
| HLA-A | rs1136697 | chr6:29911069-29911069 |
| HLA-A | rs199474490 | chr6:29911077-29911077 |
| HMOX1 | rs2071747 | chr22:35777185-35777185 |
| HP |  | chr16:72094032-72094032 |
| HP | rs201773535 | chr16:72090452-72090452 |
| HP |  | chr16:72094320-72094320 |
| HP |  | chr16:72094029-72094029 |
| IL15RA | rs145592448 | chr10:6008296-6008296 |
| IL15RA | rs148133619 | chr10:6005799-6005799 |
| IL6 |  | chr7:22766937-22766937 |
| KAT2A |  | chr17:40266241-40266241 |
| KAT2A |  | chr17:40265734-40265734 |
| KAT2A |  | chr17:40267842-40267842 |
| KCNQ1 |  | chr11:2591878-2591878 |
| LDHA | rs116841148 | chr11:18424407-18424407 |
| LEPR | rs144159890 | chr1:66075712-66075712 |
| LEPR |  | chr1:66081730-66081730 |
| LEPR | rs150936702 | chr1:66083694-66083694 |
| LEPR |  | chr1:66102401-66102401 |
| LIPC |  | chr15:58834793-58834793 |
| LIPC |  | chr15:58855765-58855765 |
| LMNA | rs6657367 | chr1:156096376-156096376 |
| LMNA | rs142000963 | chr1:156108510-156108510 |
| LPL |  | chr8:19818562-19818562 |
| LPL | rs118204057 | chr8:19811733-19811733 |
| LPL | rs118204060 | chr8:19811790-19811790 |
| LRP5 |  | chr11:68207377-68207377 |
| LRP5 |  | chr11:68190963-68190963 |
| LRP5 |  | chr11:68191023-68191023 |
| LRP5 | rs61889560 | chr11:68191036-68191036 |
| LRP5 |  | chr11:68183984-68183984 |
| LRP5 |  | chr11:68177496-68177496 |
| LRP5 |  | chr11:68181201-68181201 |
| LRP5 |  | chr11:68207263-68207263 |
| LRP5 | rs201916993 | chr11:68177526-68177526 |
| LRP5 |  | chr11:68181338-68181338 |
| LTBP4 | rs370767377 | chr19:41132912-41132912 |
| LTBP4 | rs199514354 | chr19:41135376-41135376 |
| LTBP4 | rs33937741 | chr19:41116455-41116455 |
| LTBP4 |  | chr19:41135351-41135351 |
| LTBP4 | rs35809725 | chr19:41133192-41133192 |
| LTBP4 | rs34093919 | chr19:41117300-41117300 |
| LTBP4 |  | chr19:41118062-41118062 |
| **Genes** | **SNPs ID** | **Position** |
| LTBP4 | rs201619081 | chr19:41117142-41117142 |
| LTBP4 |  | chr19:41103282-41103282 |
| LTBP4 | rs376168693 | chr19:41125371-41125371 |
| LTBP4 | rs369221693 | chr19:41128456-41128456 |
| MC4R | rs13447336 | chr18:58038829-58038829 |
| MC4R |  | chr18:58039122-58039122 |
| MC4R | rs199558727 | chr18:58039356-58039356 |
| MC4R | rs13447329 | chr18:58039248-58039248 |
| MLH1 | rs63749900 | chr3:37090087-37090087 |
| MLH1 | rs35831931 | chr3:37092019-37092019 |
| MLH1 | rs35001569 | chr3:37089130-37089130 |
| MLH1 | rs63750449 | chr3:37089131-37089131 |
| MLH1 | rs56185292 | chr3:37083821-37083821 |
| MLH1 | rs41295284 | chr3:37089098-37089098 |
| MLH1 |  | chr3:37090417-37090417 |
| MLH1 | rs63751467 | chr3:37061929-37061929 |
| MLH1 | rs2020873 | chr3:37092025-37092025 |
| MLH1 | rs63751049 | chr3:37061893-37061893 |
| MLH1 |  | chr3:37056007-37056007 |
| MSH2 |  | chr2:47672731-47672731 |
| MSH2 |  | chr2:47702358-47702358 |
| MSH2 | rs4987188 | chr2:47643457-47643457 |
| MSH2 | rs63750368 | chr2:47705493-47705493 |
| MSH2 | rs63750124 | chr2:47637301-47637301 |
| MYLK | rs35156360 | chr3:123451932-123451932 |
| MYLK |  | chr3:123471272-123471272 |
| MYLK |  | chr3:123376063-123376063 |
| MYLK |  | chr3:123428738-123428738 |
| MYLK | rs35912339 | chr3:123452836-123452836 |
| MYLK |  | chr3:123337596-123337596 |
| MYLK |  | chr3:123419707-123419707 |
| MYLK |  | chr3:123386005-123386005 |
| MYLK | rs369537401 | chr3:123471385-123471385 |
| MYLK |  | chr3:123348333-123348333 |
| MYLK |  | chr3:123348334-123348334 |
| MYLK | rs138172035 | chr3:123427717-123427717 |
| MYLK |  | chr3:123411621-123411621 |
| MYLK |  | chr3:123367890-123367890 |
| NCF4 |  | chr22:37273719-37273719 |
| NCF4 |  | chr22:37263455-37263455 |
| NCOA3 | rs6094752 | chr20:46256424-46256424 |
| NCOA3 |  | chr20:46264171-46264171 |
| NCOA3 | rs138733364 | chr20:46279918-46279918 |
| NCOA3 | rs200806631 | chr20:46279753-46279753 |
| NCOA3 | rs201832920 | chr20:46268708-46268708 |
| NCOA3 |  | chr20:46268732-46268732 |
| NCOA3 |  | chr20:46256321-46256321 |
| NCOA3 | rs200419506 | chr20:46252801-46252801 |
| NDUFA10 | rs35462421 | chr2:240951071-240951071 |
| NDUFA10 | rs144489062 | chr2:240913012-240913012 |
| **Genes** | **SNPs ID** | **Position** |
| NDUFA10 |  | chr2:240957978-240957978 |
| NDUFA10 |  | chr2:240951057-240951057 |
| NDUFA10 |  | chr2:240960659-240960659 |
| NDUFA10 | rs201842545 | chr2:240944661-240944661 |
| NDUFA10 |  | chr2:240958039-240958039 |
| NDUFA10 |  | chr2:240960712-240960712 |
| NDUFA11 |  | chr19:5893269-5893269 |
| NDUFA11 | rs202197257 | chr19:5894758-5894758 |
| NDUFA12 |  | chr12:95365338-95365338 |
| NDUFA12 |  | chr12:95397406-95397406 |
| NDUFA13,  TSSK6 |  | chr19:19626950-19626950 |
| NDUFA4 | rs150136467 | chr7:10979663-10979663 |
| NDUFA5 |  | chr7:123197142-123197142 |
| NDUFA5 | rs142377299 | chr7:123197120-123197120 |
| NDUFA5 |  | chr7:123196955-123196955 |
| NDUFA5 |  | chr7:123196936-123196936 |
| NDUFA5 | rs191194877 | chr7:123196884-123196884 |
| NDUFA5 |  | chr7:123196827-123196827 |
| NDUFA6 | rs113437301 | chr22:42482252-42482252 |
| NDUFA7 | rs12986323 | chr19:8381489-8381489 |
| NDUFA8 | rs149881897 | chr9:124914609-124914609 |
| NDUFA9 | rs34076756 | chr12:4791451-4791451 |
| NDUFA9 | rs35263902 | chr12:4763994-4763994 |
| NDUFA9 |  | chr12:4777682-4777682 |
| NDUFA9 |  | chr12:4796219-4796219 |
| NDUFAB1 |  | chr16:23596663-23596663 |
| NDUFB3 | rs144513268 | chr2:201943624-201943624 |
| NDUFB3 | rs142609245 | chr2:201943669-201943669 |
| NDUFB5 |  | chr3:179333811-179333811 |
| NDUFB5 | rs35399127 | chr3:179322703-179322703 |
| NDUFB6 | rs201078099 | chr9:32567093-32567093 |
| NDUFB7 |  | chr19:14677587-14677587 |
| NDUFB9 |  | chr8:125555393-125555393 |
| NDUFC2,  NDUFC2-KCTD14 | | chr11:77784146-77784146 |
| NDUFC2,  NDUFC2-KCTD14 | | chr11:77784147-77784147 |
| NDUFS1 |  | chr2:206997774-206997774 |
| NDUFS1 |  | chr2:207003325-207003325 |
| NDUFS2 | rs35086265 | chr1:161180482-161180482 |
| NDUFS2 |  | chr1:161179085-161179085 |
| NDUFS2 |  | chr1:161183446-161183446 |
| NDUFS3 | rs146407178 | chr11:47603682-47603682 |
| NDUFS3 |  | chr11:47600877-47600877 |
| NDUFS7 |  | chr19:1391011-1391011 |
| NDUFS8 |  | chr11:67803961-67803961 |
| NDUFS8 | rs150278938 | chr11:67799622-67799622 |
| NDUFV1 |  | chr11:67377928-67377928 |
| NDUFV1 |  | chr11:67378519-67378519 |
| NDUFV1 | rs11540012 | chr11:67375944-67375944 |
| **Genes** | **SNPs ID** | **Position** |
| NDUFV2 | rs72935225 | chr18:9122611-9122611 |
| NDUFV2 |  | chr18:9124901-9124901 |
| NDUFV2 |  | chr18:9124902-9124902 |
| NDUFV3 | rs141922962 | chr21:44317156-44317156 |
| NDUFV3 |  | chr21:44323856-44323856 |
| NDUFV3 | rs77606940 | chr21:44313454-44313454 |
| NDUFV3 | rs145030239 | chr21:44317065-44317065 |
| NFE2L2 | rs141363120 | chr2:178096406-178096406 |
| NFKB1 |  | chr4:103516106-103516106 |
| NFKB1 |  | chr4:103432092-103432092 |
| NFKB1 |  | chr4:103432093-103432093 |
| NFKB1 |  | chr4:103514626-103514626 |
| NFKB1 | rs199909759 | chr4:103537719-103537719 |
| NFKB1 | rs4648072 | chr4:103518700-103518700 |
| NHLRC2,  ADRB1 |  | chr10:115674837-115674837 |
| NHLRC2,  ADRB1 |  | chr10:115674838-115674838 |
| NHLRC2,  ADRB1 | rs186269928 | chr10:115674930-115674930 |
| NOS1 |  | chr12:117725904-117725904 |
| NOS1 | rs41356652 | chr12:117710246-117710246 |
| NOS1 | rs200972861 | chr12:117693822-117693822 |
| NOS1 | rs76090928 | chr12:117768154-117768154 |
| NOS1 |  | chr12:117693787-117693787 |
| NOS2 |  | chr17:26110047-26110047 |
| NOS2 | rs3730017 | chr17:26109102-26109102 |
| NOS2 |  | chr17:26096173-26096173 |
| NOS2 | rs28944173 | chr17:26093543-26093543 |
| NOS2 | rs149623743 | chr17:26110055-26110055 |
| NOS2 | rs148016612 | chr17:26108154-26108154 |
| NOS2 | rs201531464 | chr17:26096131-26096131 |
| NOS3 | rs368502694 | chr7:150709519-150709519 |
| NOS3 |  | chr7:150696347-150696347 |
| NOS3 |  | chr7:150696348-150696348 |
| NOS3 |  | chr7:150695677-150695677 |
| NOS3 | rs376007167 | chr7:150696369-150696369 |
| NOS3 |  | chr7:150695728-150695728 |
| NOS3 |  | chr7:150706632-150706632 |
| NOS3 |  | chr7:150704236-150704236 |
| NOS3 |  | chr7:150696378-150696378 |
| NOS3 |  | chr7:150696436-150696436 |
| NOS3 |  | chr7:150693634-150693634 |
| NOS3 |  | chr7:150693636-150693636 |
| NQO1 | rs1131341 | chr16:69748869-69748869 |
| NR1I2 |  | chr3:119526265-119526265 |
| NR1I2 | rs12721607 | chr3:119526203-119526203 |
| NR1I2 |  | chr3:119535974-119535974 |
| NR1I2 | rs12721613 | chr3:119526176-119526176 |
| NR1I2 |  | chr3:119536041-119536041 |
| NR3C1 |  | chr5:142780017-142780017 |
| **Genes** | **SNPs ID** | **Position** |
| NR3C1 |  | chr5:142780219-142780219 |
| NR3C1 |  | chr5:142680233-142680233 |
| NR3C1 |  | chr5:142675126-142675126 |
| NR3C1 |  | chr5:142779675-142779675 |
| PFKM |  | chr12:48527232-48527232 |
| PFKM |  | chr12:48527233-48527233 |
| PFKM | rs41291971 | chr12:48538908-48538908 |
| PFKM |  | chr12:48534571-48534571 |
| PFKM |  | chr12:48528047-48528047 |
| PFKM |  | chr12:48528577-48528577 |
| PFKM |  | chr12:48537832-48537832 |
| PGAM2 | rs61756062 | chr7:44104788-44104788 |
| PLCG1 |  | chr20:39797411-39797411 |
| PLCG1 |  | chr20:39802161-39802161 |
| PLCG1 |  | chr20:39794989-39794989 |
| PLCG1 |  | chr20:39794907-39794907 |
| PLCG1 |  | chr20:39798841-39798841 |
| PLCG1 |  | chr20:39802301-39802301 |
| PLCG1 |  | chr20:39792381-39792381 |
| PLCG1 |  | chr20:39801446-39801446 |
| PNMT |  | chr17:37826032-37826032 |
| PNMT | rs34530498 | chr17:37826013-37826013 |
| PNMT | rs150317672 | chr17:37826544-37826544 |
| PNMT | rs72554035 | chr17:37825998-37825998 |
| PON1 | rs144390653 | chr7:94940880-94940880 |
| PON1 | rs72552788 | chr7:94944735-94944735 |
| PPARA |  | chr22:46614173-46614173 |
| PPARA |  | chr22:46614189-46614189 |
| PPARA |  | chr22:46614190-46614190 |
| PPARG |  | chr3:12458453-12458453 |
| PPARG |  | chr3:12475493-12475493 |
| PPARG |  | chr3:12447449-12447449 |
| PPARG |  | chr3:12434185-12434185 |
| PPARG |  | chr3:12475415-12475415 |
| PPARG |  | chr3:12447503-12447503 |
| PPARGC1A |  | chr4:23826104-23826104 |
| PPARGC1A |  | chr4:23886457-23886457 |
| PPARGC1A |  | chr4:23831108-23831108 |
| PPARGC1A | rs199902625 | chr4:23816049-23816049 |
| PPARGC1A | rs148144750 | chr4:23803919-23803919 |
| PPARGC1A | rs142669571 | chr4:23886388-23886388 |
| PPARGC1A |  | chr4:23814701-23814701 |
| PRKAB2 | rs34838459 | chr1:146643580-146643580 |
| PRLHR |  | chr10:120353733-120353733 |
| PTGS2 |  | chr1:186646011-186646011 |
| PTGS2 |  | chr1:186645242-186645242 |
| PTGS2 | rs199820825 | chr1:186643727-186643727 |
| PYGM |  | chr11:64527302-64527302 |
| PYGM | rs113806080 | chr11:64518016-64518016 |
| PYGM | rs200101718 | chr11:64520571-64520571 |
| **Genes** | **SNPs ID** | **Position** |
| PYGM | rs115690781 | chr11:64522824-64522824 |
| PYGM |  | chr11:64517954-64517954 |
| PYGM | rs145881639 | chr11:64527310-64527310 |
| PYGM | rs116135678 | chr11:64521496-64521496 |
| RAC2 |  | chr22:37622838-37622838 |
| RALBP1 |  | chr18:9535674-9535674 |
| RALBP1 |  | chr18:9522338-9522338 |
| RYR2 |  | chr1:237880649-237880649 |
| RYR2 |  | chr1:237863717-237863717 |
| RYR2 |  | chr1:237824227-237824227 |
| RYR2 |  | chr1:237823367-237823367 |
| RYR2 |  | chr1:237870285-237870285 |
| RYR2 |  | chr1:237872268-237872268 |
| RYR2 |  | chr1:237872280-237872280 |
| RYR2 |  | chr1:237951416-237951416 |
| RYR2 |  | chr1:237811912-237811912 |
| RYR2 |  | chr1:237919642-237919642 |
| RYR2 |  | chr1:237659888-237659888 |
| RYR2 |  | chr1:237664127-237664127 |
| RYR2 |  | chr1:237674997-237674997 |
| RYR2 |  | chr1:237753175-237753175 |
| RYR2 |  | chr1:237659966-237659966 |
| RYR2 |  | chr1:237777706-237777706 |
| RYR2 |  | chr1:237919639-237919639 |
| RYR2 |  | chr1:237754138-237754138 |
| RYR2 |  | chr1:237880510-237880510 |
| RYR2 | rs149514924 | chr1:237711862-237711862 |
| SDHA | rs76896145 | chr5:236649-236649 |
| SDHA | rs138277996 | chr5:236676-236676 |
| SDHA | rs111387770 | chr5:236678-236678 |
| SDHA | rs201741295 | chr5:236619-236619 |
| SDHA | rs201139275 | chr5:236628-236628 |
| SDHA |  | chr5:236642-236642 |
| SDHA |  | chr5:236681-236681 |
| SDHB |  | chr1:17354355-17354355 |
| SDHB |  | chr1:17350500-17350500 |
| SDHC |  | chr1:161326623-161326623 |
| SDHC | rs201210474 | chr1:161332307-161332307 |
| SDHD | rs11214077 | chr11:111958677-111958677 |
| SDHD | rs111659413 | chr11:111963883-111963883 |
| SERPINE1 |  | chr7:100773719-100773719 |
| SERPINE1 |  | chr7:100771871-100771871 |
| SERPINE1 |  | chr7:100771872-100771872 |
| SERPINE1 |  | chr7:100771865-100771865 |
| SFTPB | rs150799880 | chr2:85892473-85892473 |
| SGCA |  | chr17:48245926-48245926 |
| SGCA |  | chr17:48245779-48245779 |
| SGCA | rs372210292 | chr17:48245758-48245758 |
| SGCA |  | chr17:48253207-48253207 |
| SGCG | rs200191311 | chr13:23894793-23894793 |
| **Genes** | **SNPs ID** | **Position** |
| SIRT1 |  | chr10:69672666-69672666 |
| SIRT1 |  | chr10:69644909-69644909 |
| SIRT3 | rs61748606 | chr11:230474-230474 |
| SIRT3 |  | chr11:218791-218791 |
| SIRT3 | rs2974901 | chr11:236306-236306 |
| SIRT3 | rs147722093 | chr11:224194-224194 |
| SIRT3 | rs202066094 | chr11:219008-219008 |
| SIRT3 |  | chr11:233037-233037 |
| SLC16A1 |  | chr1:113460371-113460371 |
| SLC16A1 | rs76000658 | chr1:113464673-113464673 |
| SLC22A16 | rs41288594 | chr6:110763935-110763935 |
| SLC22A16 |  | chr6:110778099-110778099 |
| SLC22A16 |  | chr6:110757113-110757113 |
| SLC22A16 | rs61729086 | chr6:110768128-110768128 |
| SLC22A16 | rs146329765 | chr6:110763506-110763506 |
| SLC22A16 |  | chr6:110763890-110763890 |
| SLC25A4 |  | chr4:186066165-186066165 |
| SLC28A3 |  | chr9:86912182-86912182 |
| SLC28A3 |  | chr9:86912183-86912183 |
| SOD1 |  | chr21:33040856-33040856 |
| TK2 | rs200121712 | chr16:66583871-66583871 |
| TK2 |  | chr16:66584086-66584086 |
| TNF |  | chr6:31545243-31545243 |
| TOP1MT | rs201456967 | chr8:144416967-144416967 |
| TOP1MT |  | chr8:144408466-144408466 |
| TOP1MT |  | chr8:144399938-144399938 |
| TOP1MT | rs139956930 | chr8:144411609-144411609 |
| TOP1MT |  | chr8:144398198-144398198 |
| TOP1MT | rs369663765 | chr8:144413430-144413430 |
| TOP2A |  | chr17:38564238-38564238 |
| TOP2A | rs11540720 | chr17:38545824-38545824 |
| TOP2A | rs61756342 | chr17:38559269-38559269 |
| TOP2A |  | chr17:38567619-38567619 |
| TOP2A |  | chr17:38546384-38546384 |
| TOP2B |  | chr3:25674035-25674035 |
| TOP2B |  | chr3:25666192-25666192 |
| TOP2B | rs145455403 | chr3:25705758-25705758 |
| TOP2B | rs200930765 | chr3:25666268-25666268 |
| TOP2B |  | chr3:25659978-25659978 |
| TOP2B |  | chr3:25660150-25660150 |
| TOP2B |  | chr3:25660151-25660151 |
| TOP2B | rs61751634 | chr3:25646268-25646268 |
| TOP2B | rs187350468 | chr3:25639821-25639821 |
| TOP2B |  | chr3:25674005-25674005 |
| TOP2B |  | chr3:25668084-25668084 |
| TP53 | rs34949160 | chr17:7578146-7578146 |
| TTN | rs4145333 | chr2:179444768-179444768 |
| TTN | rs72646809 | chr2:179472223-179472223 |
| TTN |  | chr2:179592096-179592096 |
| TTN |  | chr2:179597593-179597593 |
| **Genes** | **SNPs ID** | **Position** |
| TTN | rs34618570 | chr2:179605380-179605380 |
| TTN | rs6433728 | chr2:179621477-179621477 |
| TTN |  | chr2:179469738-179469738 |
| TTN | rs922985 | chr2:179615931-179615931 |
| TTN | rs1552280 | chr2:179644035-179644035 |
| TTN | rs56273463 | chr2:179400130-179400130 |
| TTN | rs149001703 | chr2:179404402-179404402 |
| TTN | rs55634791 | chr2:179428168-179428168 |
| TTN |  | chr2:179481488-179481488 |
| TTN |  | chr2:179481489-179481489 |
| TTN |  | chr2:179542464-179542464 |
| TTN |  | chr2:179549404-179549404 |
| TTN |  | chr2:179599069-179599069 |
| TTN |  | chr2:179599074-179599074 |
| TTN |  | chr2:179396320-179396320 |
| TTN | rs62621206 | chr2:179410282-179410282 |
| TTN | rs370696758 | chr2:179410954-179410954 |
| TTN | rs146181116 | chr2:179419792-179419792 |
| TTN | rs72648982 | chr2:179582853-179582853 |
| TTN |  | chr2:179590256-179590256 |
| TTN | rs17355460 | chr2:179590329-179590329 |
| TTN |  | chr2:179406294-179406294 |
| TTN |  | chr2:179415710-179415710 |
| TTN |  | chr2:179431207-179431207 |
| TTN |  | chr2:179435027-179435027 |
| TTN |  | chr2:179439880-179439880 |
| TTN |  | chr2:179462477-179462477 |
| TTN |  | chr2:179481892-179481892 |
| TTN |  | chr2:179596191-179596191 |
| TTN |  | chr2:179598004-179598004 |
| TTN | rs72647870 | chr2:179645962-179645962 |
| TTN |  | chr2:179399748-179399748 |
| TTN |  | chr2:179575869-179575869 |
| TTN | rs16866391 | chr2:179414162-179414162 |
| TTN | rs140319117 | chr2:179399071-179399071 |
| TTN | rs55675869 | chr2:179401740-179401740 |
| TTN | rs72648272 | chr2:179404550-179404550 |
| TTN | rs72648257 | chr2:179411011-179411011 |
| TTN | rs3731745 | chr2:179431594-179431594 |
| TTN | rs56018860 | chr2:179433221-179433221 |
| TTN | rs3813246 | chr2:179433580-179433580 |
| TTN | rs3813245 | chr2:179434139-179434139 |
| TTN | rs2303832 | chr2:179472292-179472292 |
| TTN | rs2288563 | chr2:179499530-179499530 |
| TTN | rs72650011 | chr2:179567340-179567340 |
| TTN | rs2562832 | chr2:179581835-179581835 |
| TTN | rs2742347 | chr2:179600648-179600648 |
| TTN | rs1883085 | chr2:179604160-179604160 |
| TTN | rs2562829 | chr2:179604366-179604366 |
| TTN | rs66677602 | chr2:179611711-179611711 |
| **Genes** | **SNPs ID** | **Position** |
| TTN | rs72647894 | chr2:179632598-179632598 |
| TTN | rs2306636 | chr2:179634936-179634936 |
| TTN | rs56142888 | chr2:179637861-179637861 |
| TTN | rs141213991 | chr2:179641112-179641112 |
| TTN | rs12476289 | chr2:179641975-179641975 |
| TTN | rs68080670 | chr2:179396766-179396766 |
| TTN |  | chr2:179415859-179415859 |
| TTN | rs72646869 | chr2:179446381-179446381 |
| TTN | rs141973925 | chr2:179455352-179455352 |
| TTN | rs13398235 | chr2:179578704-179578704 |
| TTN | rs11888217 | chr2:179593352-179593352 |
| TTN | rs4893853 | chr2:179632496-179632496 |
| TTN | rs4894048 | chr2:179638721-179638721 |
| TTN | rs72646850 | chr2:179453429-179453429 |
| TTN | rs116676813 | chr2:179549131-179549131 |
| TTN | rs16866378 | chr2:179393111-179393111 |
| TTN | rs16866380 | chr2:179395560-179395560 |
| TTN | rs3731752 | chr2:179398509-179398509 |
| TTN | rs3813243 | chr2:179434516-179434516 |
| TTN | rs72646861 | chr2:179449186-179449186 |
| TTN | rs16866412 | chr2:179474668-179474668 |
| TTN | rs72650066 | chr2:179515483-179515483 |
| TTN | rs72650034 | chr2:179547465-179547465 |
| TTN | rs36021856 | chr2:179643775-179643775 |
| TTN | rs375407842 | chr2:179428769-179428769 |
| TTN | rs373353480 | chr2:179458396-179458396 |
| TTN |  | chr2:179614454-179614454 |
| TTN |  | chr2:179458020-179458020 |
| TTN |  | chr2:179576757-179576757 |
| TTN | rs200165636 | chr2:179666894-179666894 |
| TTN |  | chr2:179396329-179396329 |
| TTN |  | chr2:179431444-179431444 |
| TTN |  | chr2:179433317-179433317 |
| TTN | rs33917087 | chr2:179634961-179634961 |
| TTN |  | chr2:179415776-179415776 |
| TTN | rs56307213 | chr2:179431076-179431076 |
| TTN |  | chr2:179439877-179439877 |
| TTN |  | chr2:179481894-179481894 |
| TTN | rs72650006 | chr2:179569387-179569387 |
| TTN | rs72648987 | chr2:179579977-179579977 |
| TTN |  | chr2:179584430-179584430 |
| TTN | rs56308529 | chr2:179396162-179396162 |
| TTN | rs55742743 | chr2:179401742-179401742 |
| TTN |  | chr2:179425213-179425213 |
| TTN |  | chr2:179572274-179572274 |
| TTN |  | chr2:179579273-179579273 |
| TTN |  | chr2:179647782-179647782 |
| TTN |  | chr2:179647783-179647783 |
| TTN | rs2278196 | chr2:179400895-179400895 |
| TTN | rs72648270 | chr2:179404628-179404628 |
| **Genes** | **SNPs ID** | **Position** |
| TTN | rs72648273 | chr2:179404498-179404498 |
| TTN | rs199895260 | chr2:179454530-179454530 |
| TTN | rs72648913 | chr2:179610967-179610967 |
| TTN |  | chr2:179623887-179623887 |
| TTN |  | chr2:179664292-179664292 |
| TTN | rs55880440 | chr2:179393859-179393859 |
| TTN | rs34070843 | chr2:179605725-179605725 |
| TTN | rs201080904 | chr2:179406266-179406266 |
| TTN | rs200144345 | chr2:179439823-179439823 |
| TTN | rs376800688 | chr2:179452459-179452459 |
| TTN |  | chr2:179587097-179587097 |
| TTN | rs72648942 | chr2:179596554-179596554 |
| TTN | rs72677232 | chr2:179482937-179482937 |
| TTN | rs72648970 | chr2:179586604-179586604 |
| TTN | rs55980498 | chr2:179441932-179441932 |
| TTN |  | chr2:179454392-179454392 |
| TTN |  | chr2:179454393-179454393 |
| TTN |  | chr2:179595277-179595277 |
| TTN | rs72648263 | chr2:179410184-179410184 |
| TTN | rs72648240 | chr2:179417919-179417919 |
| TTN |  | chr2:179428678-179428678 |
| TTN |  | chr2:179407159-179407159 |
| TTN |  | chr2:179517220-179517220 |
| TTN |  | chr2:179523496-179523496 |
| TTN |  | chr2:179422882-179422882 |
| TTN | rs36043230 | chr2:179477267-179477267 |
| TTN |  | chr2:179413601-179413601 |
| TTN |  | chr2:179597443-179597443 |
| TTN |  | chr2:179597444-179597444 |
| TTN |  | chr2:179424514-179424514 |
| TTN |  | chr2:179599491-179599491 |
| TTN |  | chr2:179416924-179416924 |
| TTN |  | chr2:179416925-179416925 |
| TTN |  | chr2:179434508-179434508 |
| TTN |  | chr2:179453355-179453355 |
| TTN |  | chr2:179476841-179476841 |
| TTN |  | chr2:179558686-179558686 |
| TTN |  | chr2:179644012-179644012 |
| TTN |  | chr2:179407236-179407236 |
| TTN |  | chr2:179597276-179597276 |
| TTN |  | chr2:179603994-179603994 |
| TTN |  | chr2:179622464-179622464 |
| TTN |  | chr2:179425819-179425819 |
| TTN |  | chr2:179455086-179455086 |
| TTN | rs72646885 | chr2:179441295-179441295 |
| TTN | rs72646880 | chr2:179442784-179442784 |
| TTN | rs188185141 | chr2:179462367-179462367 |
| TTN | rs72677237 | chr2:179482089-179482089 |
| TTN | rs140909116 | chr2:179613191-179613191 |
| TTN | rs13021201 | chr2:179449131-179449131 |
| **Genes** | **SNPs ID** | **Position** |
| TTN | rs72647851 | chr2:179658175-179658175 |
| TTN | rs55886356 | chr2:179399576-179399576 |
| TTN |  | chr2:179413223-179413223 |
| TTN | rs201381085 | chr2:179442198-179442198 |
| TTN |  | chr2:179616458-179616458 |
| TTN |  | chr2:179413483-179413483 |
| TTN |  | chr2:179398914-179398914 |
| TTN | rs72648245 | chr2:179414512-179414512 |
| TTN |  | chr2:179666979-179666979 |
| TTN |  | chr2:179403702-179403702 |
| TTN |  | chr2:179403703-179403703 |
| TTN | rs185002960 | chr2:179427796-179427796 |
| TTN |  | chr2:179464572-179464572 |
| TTN |  | chr2:179431214-179431214 |
| TTN |  | chr2:179579989-179579989 |
| TTN |  | chr2:179579990-179579990 |
| TTN |  | chr2:179399913-179399913 |
| TTN |  | chr2:179419338-179419338 |
| TTN |  | chr2:179498271-179498271 |
| TTN |  | chr2:179603970-179603970 |
| TTN | rs72677231 | chr2:179482994-179482994 |
| TTN | rs201213901 | chr2:179472662-179472662 |
| TTN | rs368069666 | chr2:179425787-179425787 |
| TTN |  | chr2:179455128-179455128 |
| TTN | rs67665715 | chr2:179412452-179412452 |
| TTN | rs747122 | chr2:179414318-179414318 |
| TTN | rs11887722 | chr2:179416659-179416659 |
| TTN | rs11896637 | chr2:179428061-179428061 |
| TTN | rs79926414 | chr2:179430224-179430224 |
| TTN | rs744427 | chr2:179435887-179435887 |
| TTN | rs72646881 | chr2:179441917-179441917 |
| TTN | rs62618736 | chr2:179447787-179447787 |
| TTN | rs73038324 | chr2:179560847-179560847 |
| TTN | rs146496197 | chr2:179640923-179640923 |
| TTN | rs72650031 | chr2:179549407-179549407 |
| TTN | rs4894028 | chr2:179403750-179403750 |
| TTN | rs62621236 | chr2:179408713-179408713 |
| TTN | rs10164753 | chr2:179438866-179438866 |
| TTN | rs201412693 | chr2:179468704-179468704 |
| TTN | rs4893852 | chr2:179571448-179571448 |
| TTN | rs17355446 | chr2:179589241-179589241 |
| TTN | rs72648247 | chr2:179414177-179414177 |
| TTN | rs72648929 | chr2:179599473-179599473 |
| TTN |  | chr2:179440885-179440885 |
| TTN | rs72648984 | chr2:179582760-179582760 |
| TTN | rs72648930 | chr2:179598553-179598553 |
| TTN |  | chr2:179638651-179638651 |
| TTN |  | chr2:179641901-179641901 |
| TTN |  | chr2:179439491-179439491 |
| TTN |  | chr2:179447927-179447927 |
| **Genes** | **SNPs ID** | **Position** |
| TTN |  | chr2:179476557-179476557 |
| TTN | rs72650019 | chr2:179559353-179559353 |
| TTN |  | chr2:179605073-179605073 |
| TTN | rs72647897 | chr2:179615278-179615278 |
| TTN |  | chr2:179419464-179419464 |
| TTN |  | chr2:179595241-179595241 |
| TTN | rs72647876 | chr2:179642589-179642589 |
| TTN | rs35683768 | chr2:179666982-179666982 |
| TTN |  | chr2:179501444-179501444 |
| TTN | rs200771189 | chr2:179429387-179429387 |
| TTN | rs72648925 | chr2:179600303-179600303 |
| TTN |  | chr2:179469873-179469873 |
| TTN |  | chr2:179593664-179593664 |
| TTN | rs34819099 | chr2:179628918-179628918 |
| TTN | rs375861551 | chr2:179404393-179404393 |
| TTN | rs55866005 | chr2:179396782-179396782 |
| TTN | rs72677225 | chr2:179486037-179486037 |
| TTN | rs200815663 | chr2:179528017-179528017 |
| TTN | rs72648227 | chr2:179422669-179422669 |
| TTN |  | chr2:179616446-179616446 |
| TTN | rs371249764 | chr2:179631128-179631128 |
| TTN | rs66961115 | chr2:179395554-179395554 |
| TTN | rs67254537 | chr2:179395555-179395555 |
| TTN | rs56324595 | chr2:179395573-179395573 |
| TTN |  | chr2:179404872-179404872 |
| TTN |  | chr2:179427754-179427754 |
| TTN | rs72648937 | chr2:179597600-179597600 |
| TTN |  | chr2:179599144-179599144 |
| TTN |  | chr2:179604042-179604042 |
| TTN |  | chr2:179622616-179622616 |
| TTN |  | chr2:179631233-179631233 |
| TTN | rs115150240 | chr2:179396928-179396928 |
| TTN |  | chr2:179621203-179621203 |
| TTN | rs72955213 | chr2:179621503-179621503 |
| TTN |  | chr2:179637908-179637908 |
| TTN |  | chr2:179396682-179396682 |
| TTN |  | chr2:179439884-179439884 |
| TTN | rs72629787 | chr2:179396965-179396965 |
| TTN | rs56376197 | chr2:179399539-179399539 |
| TTN | rs184643087 | chr2:179427560-179427560 |
| TTN | rs202125813 | chr2:179445129-179445129 |
| TTN | rs72646833 | chr2:179462345-179462345 |
| TTN | rs183245562 | chr2:179485946-179485946 |
| TTN | rs57389274 | chr2:179621353-179621353 |
| TTN | rs111727915 | chr2:179419353-179419353 |
| TTN | rs186681106 | chr2:179433046-179433046 |
| TTN | rs55948748 | chr2:179448393-179448393 |
| TTN |  | chr2:179458426-179458426 |
| TTN | rs200459347 | chr2:179556748-179556748 |
| TTN | rs187925021 | chr2:179587955-179587955 |
| **Genes** | **SNPs ID** | **Position** |
| TTN | rs148164929 | chr2:179606288-179606288 |
| TTN | rs370079368 | chr2:179410397-179410397 |
| TTN |  | chr2:179596049-179596049 |
| TTN | rs116142642 | chr2:179468729-179468729 |
| TTN |  | chr2:179469468-179469468 |
| TTN | rs370367786 | chr2:179431879-179431879 |
| TTN |  | chr2:179501209-179501209 |
| TTN | rs371639583 | chr2:179659236-179659236 |
| TTN | rs368615862 | chr2:179425021-179425021 |
| TTN | rs201802447 | chr2:179468833-179468833 |
| TTN | rs3813244 | chr2:179434160-179434160 |
| TTN | rs201804005 | chr2:179437255-179437255 |
| TTN | rs138440219 | chr2:179615654-179615654 |
| TTN |  | chr2:179396446-179396446 |
| TTN |  | chr2:179409096-179409096 |
| TTN | rs55801134 | chr2:179440163-179440163 |
| TTN | rs17354992 | chr2:179486223-179486223 |
| TTN | rs17452588 | chr2:179585312-179585312 |
| TTN |  | chr2:179404501-179404501 |
| TTN |  | chr2:179410298-179410298 |
| TTN | rs72648228 | chr2:179422470-179422470 |
| TTN | rs72646849 | chr2:179454020-179454020 |
| TTN | rs200952728 | chr2:179553849-179553849 |
| TTN | rs55762754 | chr2:179437034-179437034 |
| TTN |  | chr2:179441513-179441513 |
| TTN |  | chr2:179397342-179397342 |
| TTN | rs72677243 | chr2:179479288-179479288 |
| TTN | rs140760859 | chr2:179643656-179643656 |
| TTN |  | chr2:179605706-179605706 |
| TTN |  | chr2:179605707-179605707 |
| TTN | rs369094355 | chr2:179560942-179560942 |
| TTN | rs72648972 | chr2:179585257-179585257 |
| TTN | rs72648960 | chr2:179589058-179589058 |
| TTN | rs34924609 | chr2:179399677-179399677 |
| TTN | rs201060254 | chr2:179417104-179417104 |
| TTN | rs33971253 | chr2:179605991-179605991 |
| TTN |  | chr2:179413571-179413571 |
| TTN |  | chr2:179430124-179430124 |
| TTN |  | chr2:179641283-179641283 |
| TTN |  | chr2:179642153-179642153 |
| TTN | rs72648927 | chr2:179599667-179599667 |
| TTN | rs371818894 | chr2:179439710-179439710 |
| TTN | rs376039623 | chr2:179408585-179408585 |
| TTN | rs115658240 | chr2:179441119-179441119 |
| TTN | rs368607833 | chr2:179528603-179528603 |
| TTN |  | chr2:179395508-179395508 |
| TTN | rs201512527 | chr2:179435964-179435964 |
| TTN | rs75031300 | chr2:179638834-179638834 |
| TTN | rs376015894 | chr2:179659795-179659795 |
| TTN |  | chr2:179399755-179399755 |
| **Genes** | **SNPs ID** | **Position** |
| TTN | rs72648244 | chr2:179414992-179414992 |
| TTN |  | chr2:179446764-179446764 |
| TTN | rs55704830 | chr2:179406044-179406044 |
| TTN | rs373129706 | chr2:179425387-179425387 |
| TTN |  | chr2:179456911-179456911 |
| TTN |  | chr2:179547573-179547573 |
| TTN |  | chr2:179448493-179448493 |
| TTN |  | chr2:179400541-179400541 |
| TTN |  | chr2:179464119-179464119 |
| TTN | rs201717871 | chr2:179483108-179483108 |
| TTN | rs149470241 | chr2:179590714-179590714 |
| TTN |  | chr2:179396961-179396961 |
| TTN | rs369671334 | chr2:179438528-179438528 |
| TTN | rs375000725 | chr2:179463987-179463987 |
| TTN | rs55837610 | chr2:179439154-179439154 |
| TTN | rs377000174 | chr2:179463490-179463490 |
| TTN |  | chr2:179629367-179629367 |
| TTN |  | chr2:179439974-179439974 |
| TTN |  | chr2:179471766-179471766 |
| TTN |  | chr2:179633472-179633472 |
| TTN |  | chr2:179640399-179640399 |
| TTN |  | chr2:179649074-179649074 |
| TTN |  | chr2:179649075-179649075 |
| TTN |  | chr2:179435250-179435250 |
| TTN | rs72647843 | chr2:179665163-179665163 |
| TTN |  | chr2:179604221-179604221 |
| TTN |  | chr2:179604222-179604222 |
| TTN |  | chr2:179605658-179605658 |
| TTN | rs72629783 | chr2:179398465-179398465 |
| TTN |  | chr2:179419203-179419203 |
| TTN |  | chr2:179436320-179436320 |
| TTN |  | chr2:179666988-179666988 |
| TTN |  | chr2:179417925-179417925 |
| TTN |  | chr2:179477678-179477678 |
| TTN |  | chr2:179596838-179596838 |
| TTN | rs374605213 | chr2:179640946-179640946 |
| TTN |  | chr2:179430815-179430815 |
| TTN | rs72648989 | chr2:179578891-179578891 |
| TTN |  | chr2:179610320-179610320 |
| TTN | rs368502650 | chr2:179483349-179483349 |
| TTN |  | chr2:179587094-179587094 |
| TTN |  | chr2:179628946-179628946 |
| TTN |  | chr2:179445189-179445189 |
| TTN |  | chr2:179476584-179476584 |
| TTN |  | chr2:179497316-179497316 |
| TTN |  | chr2:179454299-179454299 |
| TTN | rs372968732 | chr2:179431544-179431544 |
| TTN | rs146983095 | chr2:179595372-179595372 |
| TTN |  | chr2:179418507-179418507 |
| TTN |  | chr2:179464030-179464030 |
| **Genes** | **SNPs ID** | **Position** |
| TTN |  | chr2:179474216-179474216 |
| TTN |  | chr2:179598041-179598041 |
| TTN | rs377095491 | chr2:179456929-179456929 |
| TTN |  | chr2:179481337-179481337 |
| TTN | rs373378672 | chr2:179600640-179600640 |
| TTN | rs373298007 | chr2:179495039-179495039 |
| TTN |  | chr2:179398944-179398944 |
| TTN | rs55842557 | chr2:179393691-179393691 |
| TTN |  | chr2:179642440-179642440 |
| TTN |  | chr2:179642441-179642441 |
| TTN |  | chr2:179430521-179430521 |
| TTN |  | chr2:179560789-179560789 |
| TTN |  | chr2:179452911-179452911 |
| TTN |  | chr2:179498789-179498789 |
| TTN |  | chr2:179621309-179621309 |
| TTN |  | chr2:179425136-179425136 |
| TTN |  | chr2:179452902-179452902 |
| TTN | rs185887755 | chr2:179430305-179430305 |
| TTN | rs56372592 | chr2:179434120-179434120 |
| TTN |  | chr2:179557242-179557242 |
| TTN |  | chr2:179569098-179569098 |
| TTN | rs72649002 | chr2:179571595-179571595 |
| TTN | rs72648939 | chr2:179597259-179597259 |
| TTN |  | chr2:179654142-179654142 |
| TTN |  | chr2:179659911-179659911 |
| TTN |  | chr2:179472610-179472610 |
| TTN | rs111671438 | chr2:179563606-179563606 |
| TTN | rs56201325 | chr2:179437928-179437928 |
| TTN |  | chr2:179572519-179572519 |
| TTN | rs74580375 | chr2:179486431-179486431 |
| TTN | rs374620001 | chr2:179635985-179635985 |
| TTN |  | chr2:179599088-179599088 |
| TTN |  | chr2:179499914-179499914 |
| TTN | rs72646873 | chr2:179444915-179444915 |
| TTN |  | chr2:179596920-179596920 |
| TTN |  | chr2:179453918-179453918 |
| TTN |  | chr2:179468804-179468804 |
| TTN | rs185913848 | chr2:179472293-179472293 |
| TTN | rs189951108 | chr2:179588279-179588279 |
| TTN |  | chr2:179600300-179600300 |
| TTN |  | chr2:179438560-179438560 |
| TTN | rs376823283 | chr2:179579172-179579172 |
| TTN |  | chr2:179481885-179481885 |
| TTN |  | chr2:179486283-179486283 |
| TTN |  | chr2:179571618-179571618 |
| TTN | rs72646808 | chr2:179472319-179472319 |
| TTN |  | chr2:179669295-179669295 |
| TTN | rs202064385 | chr2:179407482-179407482 |
| TTN | rs199590524 | chr2:179659806-179659806 |
| TTN |  | chr2:179410607-179410607 |
| **Genes** | **SNPs ID** | **Position** |
| TTN | rs202014478 | chr2:179537200-179537200 |
| TTN | rs181104321 | chr2:179412307-179412307 |
| TTN | rs200854704 | chr2:179414944-179414944 |
| TTN | rs201348580 | chr2:179426711-179426711 |
| TTN |  | chr2:179460495-179460495 |
| TTN | rs201284459 | chr2:179639725-179639725 |
| TTN |  | chr2:179428595-179428595 |
| TTN |  | chr2:179442825-179442825 |
| TTN |  | chr2:179485125-179485125 |
| TTN | rs376537509 | chr2:179414062-179414062 |
| TTN |  | chr2:179438390-179438390 |
| TTN | rs200213832 | chr2:179554549-179554549 |
| TTN |  | chr2:179465827-179465827 |
| TTN |  | chr2:179430142-179430142 |
| TTN |  | chr2:179430960-179430960 |
| TTN |  | chr2:179457370-179457370 |
| TTN |  | chr2:179484965-179484965 |
| TTN |  | chr2:179400433-179400433 |
| TTN |  | chr2:179404661-179404661 |
| TTN |  | chr2:179460521-179460521 |
| TTN | rs72648953 | chr2:179593503-179593503 |
| UCP2 | rs201315561 | chr11:73689345-73689345 |
| UCP2 |  | chr11:73687690-73687690 |
| UCP3 | rs377428955 | chr11:73717376-73717376 |
| UCP3 | rs143786748 | chr11:73717340-73717340 |
| UCP3 |  | chr11:73712523-73712523 |
| UCP3 |  | chr11:73712464-73712464 |
| UCP3 | rs2229707 | chr11:73717247-73717247 |
| UCP3 | rs139405752 | chr11:73712513-73712513 |
| UGT1A1,  UGT1A10,  UGT1A3,  UGT1A4,  UGT1A5,  UGT1A6,  UGT1A7,  UGT1A8,  UGT1A9 |  | chr2:234677027-234677027 |
| UGT1A1,  UGT1A10,  UGT1A3,  UGT1A4,  UGT1A5,  UGT1A6,  UGT1A7,  UGT1A8,  UGT1A9 |  | chr2:234680986-234680986 |

| **Genes** | **SNPs ID** | **Position** |
| --- | --- | --- |
| UGT1A1,  UGT1A10,  UGT1A3,  UGT1A4,  UGT1A5,  UGT1A6,  UGT1A7,  UGT1A8,  UGT1A9 |  | chr2:234680917-234680917 |
| UGT1A6 |  | chr2:234602507-234602507 |
| UGT1A6 |  | chr2:234601816-234601816 |
| UGT1A6 |  | chr2:234601817-234601817 |
| ULK1 |  | chr12:132394335-132394335 |
| ULK1 |  | chr12:132393243-132393243 |
| ULK1 |  | chr12:132403898-132403898 |
| ULK1 | rs34936984 | chr12:132402007-132402007 |
| VDR |  | chr12:48249552-48249552 |
| VDR | rs78942213 | chr12:48238711-48238711 |
| VEGFA |  | chr6:43748483-43748483 |
| VEGFA |  | chr6:43745336-43745336 |
| VEGFA |  | chr6:43738507-43738507 |
| VEGFA |  | chr6:43745283-43745283 |
| VEGFA |  | chr6:43748497-43748497 |
| VEGFA |  | chr6:43748498-43748498 |
| XDH | rs45564939 | chr2:31560572-31560572 |
| XDH | rs17011368 | chr2:31590917-31590917 |
| XDH |  | chr2:31562390-31562390 |
| XDH |  | chr2:31565072-31565072 |
| XDH |  | chr2:31590859-31590859 |
| XDH | rs138649664 | chr2:31600072-31600072 |
| XDH | rs148585342 | chr2:31588857-31588857 |
| XDH | rs149487595 | chr2:31609376-31609376 |
| XDH | rs146283435 | chr2:31570459-31570459 |
| XDH | rs149717617 | chr2:31588938-31588938 |
| XDH | rs201332711 | chr2:31610711-31610711 |
| XDH | rs201810251 | chr2:31605901-31605901 |
| XDH |  | chr2:31560536-31560536 |

Supplemental Digital Content, Table S3. Clinical characteristics of acute lymphoblastic leukemia survivors with a very low or very high cardiorespiratory fitness level

|  | Group 2 | |
| --- | --- | --- |
|  | **≤48.3%** | **≥125.6%** |
| Total | 5 | 5 |
| Gender (Females / Males) | 3/2 | 3/2 |
| Age at visit (y) | 20.0 ± 2.4 | 20.0 ± 7.2 |
| Age at cancer diagnosis (y) | 3.8 ± 3.6 | 6.4 ± 5.5 |
| Time from the end of the treatment (y) | 15.3 ± 2.5 | 10.9 ± 3.7 |
| Weight (kg) | 58.7 ± 24.2 | 65.0 ± 17.5 |
| Height (cm) | 165.7 ± 11.6 | 165.5 ± 14.1 |
| $\dot{\text{V}}$O_2_ peak (mL.kg^-1^.min^-1^) | 19.7 ± 4.8 | 42.7 ± 2.9* |
| Power at $\dot{\text{V}}$O_2_ peak (W) | 110.0 ± 65.2 | 189.8 ± 9.2* |
| $\dot{\text{V}}$O_2_ peak predicted (%) | 43.5 ± 4.7 | 225.0 ± 88.4* |
| Physical activity (min) | 14.7 ± 20.2 | 128.0 ± 1.9* |

Physical activity variable represents minutes per day of moderate or intense leisure physical activities. Data are expressed as percentages or as means ± SD. **p* < .05

**Supplemental Digital Content, Table S4. Significant genetic associations between very low or very high cardiorespiratory fitness and common variants**

|  | Gene | SNPs ID | Minor Alleles | |  | Major Alleles | | p-value | FDR | MAF | OR (95% CI) |  |  |
| --- | --- | --- | --- | --- | --- | --- | --- | --- | --- | --- | --- | --- | --- |
|  |  |  | **Affected,**  **N (%)** | **Unaffected,**  **N (%)** |  | **Affected,**  **N (%)** | **Unaffected,**  **N (%)** |  |  |  |  |  |  |
| Survivors ≤48.3% predicted $\dot{\text{V}}$O2 peak | IGFBP1 | rs4619 | 1 (10.00) | 9 (90.00) |  | 8 (80.00) | 2 (20.00) | 0.005 | 0.08 | 0.39 | 0.03 (0.002 - 0.37) |  |  |
| Female survivors ≤48.3% predicted $\dot{\text{V}}$O2 peak | LEPR | rs1137100 | 0 (0.00) | 8 (100.00) |  | 5 (83.33) | 1 (16.67) | 0.002 | 0.09 | 0.25 | - |  |  |
|  | IGFBP1 | rs4619 | 1 (12.50) | 7 (87.50) |  | 6 (100.00) | 0 (0.00) | 0.004 | 0.09 | 0.42 | - |  |  |

SNPs, single-nucleotide polymorphisms; MAF, Minor allele frequency; OR, Odds Ratio. Threshold of FDR (false discovery rate) was 0.10. Affected were survivors who have a cardiorespiratory fitness level ≥125.6% and unaffected were survivors who have a cardiorespiratory fitness level ≤48.3%. Results from genetic associations were presented using allelic model.

**Supplemental Digital Content, Table S5. Significant genetic associations between cardiorespiratory fitness and rare variants**

|  | **Gene** | **Position** | **SNP ID** | **MAF** | **p-value** | **FDR** | **OR (95% CI)^a^** |
| --- | --- | --- | --- | --- | --- | --- | --- |
| HR group <83.8% predicted $\dot{\text{V}}$O2 peak | SLC22A16 | chr6:110763935-110763935 | rs41288594 | 0.008 | 0.001 | 0.04 | 2.09 (0.59 – 7.39) |
|  | **SLC22A16** | **chr6:110768128-110768128** | **rs61729086** | 0.004 |  |  |  |
|  | SLC22A16 | chr6:110763506-110763506 | rs146329765 | 0.002 |  |  |  |

HR, high risk without dexrazoxane; SNPs, single-nucleotide polymorphisms; OR, Odds ratio. Threshold of FDR (false discovery rate) was 0.10. Results from genetic associations were presented using allelic model, while results from logistic regression were presented using the genotypic model (i.e. additive). ^a^OR of significant combination, combination include all variants. Individual contribution of variant rs61729086 (noted in bold) was identified with OR=3.17 (0.51-19.85), p=0.001. The significant genetic variant rs61729086 was analyzed in a logistic regression adjusted for age at visit, sex and time from end of the treatment with OR=3.24 (0.36-29.18), p=0.29.
